# Supplementary material for: Effect of Nuts Combined with Energy Restriction on the Obesity Treatment: A Systematic Review and Meta-Analysis of Randomized Controlled Trials
Source: Foods. 2024 Sep 23;13(18):3008. doi: 10.3390/foods13183008 (PMC11431603; doi:10.3390/foods13183008)
Supplement: Supplementary file 1 [file foods-13-03008-s001.zip › foods-3180019-supplementary.pdf]

Effect of nuts combined with energy restriction on the obesity treatment: a systematic review and meta-analysis of randomized controlled trials

Darlene Larissa de Souza Vilela [1,2], Alessandra da Silva [3,4] Ana Claudia Pelissari Kravchychyn [1,2], Josefina Bressan [1,2] and Helen Hermana Miranda Hermsdorff [1,2*]

1 Laboratory of Clinical Analysis and Genomics, Department of Nutrition and Health, Universidade Federal de Viçosa (UFV), Viçosa, 36570-900, MG, Brazil; darlene.vilela@ufv.br (D.V.); ana.pelissari@ufv.br (A.C.P.K.); jbrm@ufv.br (J.B.)

2 Laboratory of Energy Metabolism and Body Composition, Department of Nutrition and Health, Universidade Federal de Viçosa (UFV), Viçosa, 36570-900, MG, Brazil.;

3 Laboratory of Health and Environment Education, Instituto Oswaldo Cruz, Rio de Janeiro, 21040-360, RJ, Brazil; alessan.drasg94@gmail.com

4 Department of Epidemiology and Quantitative Methods in Health, Escola Nacional de Saúde Pública, Fundação Oswaldo Cruz, Rio de Janeiro 21040-900, RJ Brazil.

*Correspondence: HHM Hermsdorff, E-mail: helenhermana@ufv.br; Tel.: +55-(31)-3612-5195.

Supplementary Table S1. PRISMA 2020 Checklist

Section and Topic	Item #	Checklist item	Location where item is reported
**TITLE**			
Title	1	Identify the report as a systematic review.	#1
**ABSTRACT**			
Abstract	2	See the PRISMA 2020 for Abstracts checklist.	#2
**INTRODUCTION**			
Rationale	3	Describe the rationale for the review in the context of existing knowledge.	#3
Objectives	4	Provide an explicit statement of the objective(s) or question(s) the review addresses.	#4
**METHODS**			
Eligibility criteria	5	Specify the inclusion and exclusion criteria for the review and how studies were grouped for the syntheses.	#5 and #6
Informatio nsources	6	Specify all databases, registers, websites, organisations, reference lists and other sources searched or consulted to identify studies. Specify the date when each source was last searched or consulted.	#4
Search strategy	7	Present the full search strategies for all databases, registers and websites, including any filters and limits used.	#5
Selection process	8	Specify the methods used to decide whether a study met the inclusion criteria of the review, including how many reviewers screened each recordand each report retrieved, whether they worked independently, and if applicable, details of automation tools used in the process.	#5
Data collection process	9	Specify the methods used to collect data from reports, including how many reviewers collected data from each report, whether they worked independently, any processes for obtaining or confirming data from study investigators, and if applicable, details of automation tools used in theprocess.	#5
Data items	10a	List and define all outcomes for which data were sought. Specify whether all results that were compatible with each outcome domain in each study were sought (e.g. for all measures, time points, analyses), and if not, the methods used to decide which results to collect.	#6 and 7
	10b	List and define all other variables for which data were sought (e.g. participant and intervention characteristics, funding sources). Describe any assumptions made about any missing or unclear information.	#6 and 7
Study risk of bias assessment	11	Specify the methods used to assess risk of bias in the included studies, including details of the tool(s) used, how many reviewers assessed each study and whether they worked independently, and if applicable, details of automation tools used in the process.	#7
Effect measures	12	Specify for each outcome the effect measure(s) (e.g. risk ratio, mean difference) used in the synthesis or presentation of results.	#6
Synthesis methods	13a	Describe the processes used to decide which studies were eligible for each synthesis (e.g. tabulating the study intervention characteristics and comparing against the planned groups for each synthesis (item #5)).	#6 and 7
	13b	Describe any methods required to prepare the data for presentation or synthesis, such as handling of missing summary statistics, or data conversions.	#6 and 7
	13c	Describe any methods used to tabulate or visually display results of individual studies and syntheses.	#6 and 7
	13d	Describe any methods used to synthesize results and provide a rationale for the choice(s). If meta-analysis was performed, describe themodel(s), method(s) to identify the presence and extent of statistical heterogeneity, and software package(s) used.	#6 and 7
	13e	Describe any methods used to explore possible causes of heterogeneity among study results (e.g. subgroup analysis, meta-regression).	#6 and 7
	13f	Describe any sensitivity analyses conducted to assess robustness of the synthesized results.	#6 and 7
Reporting bias assessment	14	Describe any methods used to assess risk of bias due to missing results in a synthesis (arising from reporting biases).	#6 and 7

Certainty assessment	15	Describe any methods used to assess certainty (or confidence) in the body of evidence for an outcome.	#6 and 7

Section and Topic	Item#	Checklist item	Location where item is reported
**RESULTS**			
Study selection	16a	Describe the results of the search and selection process, from the number of records identified in the search to the number of studies included inthe review, ideally using a flow diagram.	#5 and 6
	16b	Cite studies that might appear to meet the inclusion criteria, but which were excluded, and explain why they were excluded.	#5 and 6
Study characteristics	17	Cite each included study and present its characteristics.	#8-12
Risk of bias in studies	18	Present assessments of risk of bias for each included study.	#7 and 8
Results of individual studies	19	For all outcomes, present, for each study: (a) summary statistics for each group (where appropriate) and (b) an effect estimate and its precision(e.g. confidence/credible interval), ideally using structured tables or plots.	15-20
Results of syntheses	20a	For each synthesis, briefly summarise the characteristics and risk of bias among contributing studies.	#12 and 13
	20b	Present results of all statistical syntheses conducted. If meta-analysis was done, present for each the summary estimate and its precision (e.g.confidence/credible interval) and measures of statistical heterogeneity. If comparing groups, describe the direction of the effect.	#12-25
	20c	Present results of all investigations of possible causes of heterogeneity among study results.	#12-25
	20d	Present results of all sensitivity analyses conducted to assess the robustness of the synthesized results.	#12-25
Reporting biases	21	Present assessments of risk of bias due to missing results (arising from reporting biases) for each synthesis assessed.	#12-25
Certainty ofevidence	22	Present assessments of certainty (or confidence) in the body of evidence for each outcome assessed.	#12-25
**DISCUSSION**			
Discussion	23a	Provide a general interpretation of the results in the context of other evidence.	#12-15
	23b	Discuss any limitations of the evidence included in the review.	#15
	23c	Discuss any limitations of the review processes used.	#15
	23d	Discuss implications of the results for practice, policy, and future research.	#15
**OTHER INFORMATION**			
Registration and protocol	24a	Provide registration information for the review, including register name and registration number, or state that the review was not registered.	#4
	24b	Indicate where the review protocol can be accessed, or state that a protocol was not prepared.	#4
	24c	Describe and explain any amendments to information provided at registration or in the protocol.	
Support	25	Describe sources of financial or non-financial support for the review, and the role of the funders or sponsors in the review.	#16
Competing interests	26	Declare any competing interests of review authors.	#16

Availability of data, code and other materials	27	Report which of the following are publicly available and where they can be found: template data collection forms; data extracted from includedstudies; data used for all analyses; analytic code; any other materials used in the review.	#16

From: Page MJ, McKenzie JE, Bossuyt PM, Boutron I, Hoffmann TC, Mulrow CD, et al. The PRISMA 2020 statement: an updated guideline for reporting systematic reviews. BMJ 2021;372:n71. doi: 10.1136/bmj.n7 For more information, visit: http://www.prisma-statement.org/

Supplementary Table S2. The central question of the systematic review defined using the anagram PICOS

Parameter	Abbreviation	Inclusion criteria
Population	P	Adults and seniors ≥18 years old
Intervention	I	Nut consumption associated with energy restriction
Comparison	C	People on energy restriction who do not consume nuts
Outcome	O	Weight loss, changes in body composition, modification in cardiometabolic risk markers
Study design	S	Randomized controlled clinical trials

Supplementary Table S3. Complete literature search

**SCOPUS 77 RECOVERED QUOTES**
TITLE-ABS-KEY ("Caloric Restriction" OR "Restriction, Caloric" OR "Calorie Restricted Diet" OR "Calorie Restricted Diets" OR "Diet, Calorie Restricted" OR "Restricted Diet, Calorie" OR "Caloric Restricted" OR "Restricted, Caloric" OR "Low-Calorie Diet" OR "Diet, Low-Calorie" OR "Low Calorie Diet" OR "Low-Calorie Diets" AND "Weight Loss" OR "Loss, Weight" OR "Losses, Weight" OR "Weight Losses" OR "Weight Reduction" OR "Reduction, Weight" OR "Reductions, Weight" OR "Weight Reductions" AND "Nuts" OR "Nut" OR "Sweet Almond" OR "Almonds" OR "Almond" OR "Brazil Nuts" OR "Brazil Nut" OR "Nut, Brazil" OR "Cashew" OR "Cashews" OR "Filberts" OR "Filbert" OR "Hazelnuts" OR "Hazelnut" OR "Macadamia" OR "Macadamias" OR "Macadamia Nut" OR "Macadamia Nuts" OR "Pecans" OR "Pecan" OR "Hickory Nuts" OR "Hickory Nut" OR "pine nuts" OR "Pistacia vera" OR "Pistachio" OR "Pistachios" OR "Walnut" OR "Walnuts" OR "English Walnuts" OR "Juglans nigra" OR "Black Walnut" OR "Arachis hypogaea" OR "Peanuts" OR "Peanut" OR "Baru nut" OR "tree nuts" OR "groundnut")
**PUBMED 85 RECOVERED QUOTES**
((Caloric Restriction OR Restriction, Caloric OR Calorie Restricted Diet OR Calorie Restricted Diets OR Diet, Calorie Restricted OR Restricted Diet, Calorie OR Caloric Restricted OR Restricted, Caloric OR Diet, Low-Calorie OR Low Calorie Diet OR Low-Calorie Diets) AND (Weight Loss OR Loss, Weight OR Losses, Weight OR Weight Losses OR Weight Reduction OR Reduction, Weight OR Reductions, Weight OR Weight Reductions)) AND (Nuts OR Nut OR Sweet Almond OR Almonds OR Almond OR Brazil Nuts OR Brazil Nut OR Nut, Brazil OR Cashew OR Cashews OR Filberts OR Filbert OR Hazelnuts OR Hazelnut OR Macadamia OR Macadamias OR Macadamia Nut OR Macadamia Nuts OR Pecans OR Pecan OR Hickory Nuts OR Hickory Nut OR pine nuts OR Pistacia vera OR Pistachio OR Pistachios OR Walnut OR Walnuts OR English Walnuts OR Juglans nigra OR Black Walnut Arachis hypogaea OR Peanuts OR Peanut OR Baru nut OR tree nuts OR groundnut)

EMBASE 118 RECOVERED QUOTES
((((((((((caloric AND restriction OR restriction,) AND caloric OR calorie) AND restricted AND diet OR calorie) AND restricted AND diets OR diet,) AND calorie AND restricted OR restricted) AND diet, AND calorie OR caloric) AND restricted OR restricted,) AND caloric OR 'low calorie') AND diet OR diet,) AND 'low calorie' OR low) AND calorie AND diet OR 'low calorie') AND diets AND (('weight loss'/exp OR 'weight loss' OR (('weight'/exp OR weight) AND ('loss'/exp OR loss)) OR 'loss, weight' OR (loss, AND ('weight'/exp OR weight)) OR 'losses, weight' OR (losses, AND ('weight'/exp OR weight)) OR 'weight losses' OR (('weight'/exp OR weight) AND losses) OR 'weight reduction'/exp OR 'weight reduction' OR (('weight'/exp OR weight) AND ('reduction'/exp OR reduction)) OR 'reduction, weight' OR (('reduction,'/exp OR reduction,) AND ('weight'/exp OR weight)) OR 'reductions, weight' OR (reductions, AND ('weight'/exp OR weight)) OR 'weight reductions' OR (('weight'/exp OR weight) AND reductions)) AND ('nuts'/exp OR nuts) OR 'nut'/exp OR nut OR 'sweet almond'/exp OR 'sweet almond' OR (('sweet'/exp OR sweet) AND ('almond'/exp OR almond)) OR almonds OR 'almond'/exp OR almond OR 'brazil nuts' OR (('brazil'/exp OR brazil) AND ('nuts'/exp OR nuts)) OR 'brazil nut'/exp OR 'brazil nut' OR (('brazil'/exp OR brazil) AND ('nut'/exp OR nut)) OR 'nut, brazil' OR (('nut,'/exp OR nut,) AND ('brazil'/exp OR brazil)) OR cashew OR cashews OR filberts OR filbert OR hazelnuts OR 'hazelnut'/exp OR hazelnut OR 'macadamia'/exp OR macadamia OR macadamias OR 'macadamia nut' OR (('macadamia'/exp OR macadamia) AND ('nut'/exp OR nut)) OR 'macadamia nuts' OR (('macadamia'/exp OR macadamia) AND ('nuts'/exp OR nuts)) OR pecans OR 'pecan'/exp OR pecan OR 'hickory nuts' OR (hickory AND ('nuts'/exp OR nuts)) OR 'hickory nut' OR (hickory AND ('nut'/exp OR nut)) OR 'pine nuts' OR (('pine'/exp OR pine) AND ('nuts'/exp OR nuts)) OR 'pistacia vera'/exp OR 'pistacia vera' OR (('pistacia'/exp OR pistacia) AND vera) OR 'pistachio'/exp OR pistachio OR pistachios OR 'walnut'/exp OR walnut OR walnuts OR 'english walnuts' OR (('english'/exp OR english) AND walnuts) OR 'juglans nigra'/exp OR 'juglans nigra' OR (('juglans'/exp OR juglans) AND nigra) OR 'black walnut arachis hypogaea' OR (('black'/exp OR black) AND ('walnut'/exp OR walnut) AND ('arachis'/exp OR arachis) AND hypogaea) OR 'peanuts'/exp OR peanuts OR 'peanut'/exp OR peanut OR 'baru nut' OR (baru AND ('nut'/exp OR nut)) OR 'tree nuts' OR (('tree'/exp OR tree) AND ('nuts'/exp OR nuts)) OR 'groundnut'/exp OR groundnut)
**CENTRAL 118 RECOVERED QUOTES**
Caloric Restriction in Title Abstract Keyword AND "weight loss" in Title Abstract Keyword AND "nuts" in Title Abstract Keyword - (Word variations have been searched)

Supplementary Table S4. Deleted articles

**No calorie restriction (n =3)**
Godwin, N., Roberts, T., Hooshmand, S., Kern, M., & Hong, M. Y. (2019). Mixed nuts may promote satiety while maintaining stable blood glucose and insulin in healthy, obese, and overweight adults in a two-arm randomized controlled trial. *Journal of medicinal food*, *22*(4), 427-432.
Berryman, C. E., West, S. G., Fleming, J. A., Bordi, P. L., & Kris-Etherton, P. M. (2015). Effects of daily almond consumption on cardiometabolic risk and abdominal adiposity in healthy adults with elevated LDL-cholesterol: a randomized controlled trial. *Journal of the American Heart Association*, *4*(1), e000993.
Mohan, V., Gayathri, R., Jaacks, L. M., Lakshmipriya, N., Anjana, R. M., Spiegelman, D., ... & Willett, W. C. (2018). Cashew nut consumption increases HDL cholesterol and reduces systolic blood pressure in Asian Indians with type 2 diabetes: a 12-week randomized controlled trial. *The Journal of nutrition*, *148*(1), 63-69.
**Only assessed weight at baseline (n=2)**
Costa, M. A. D. C., Hermsdorff, H. H. M., Caldas, A. P. S., Rocha, D. M. U. P., da Silva, A., de Oliveira, L. L., & Bressan, J. (2021). Acute consumption of a shake containing cashew and Brazil nuts did not affect appetite in overweight subjects: a randomized, cross-over study. *European Journal of Nutrition*, *60*(8), 4321-4330.
Rosenstock, A., Connolly, M., Weller, R., & Hong, M. Y. (2020). Brazil nut consumption promotes satiety without increasing blood glucose and insulin responses in healthy adults. *Nutrire*, *45*, 1-7.
**Only assessed satiety (n=1)**
Daniela Mayumi Usuda Prado Rocha, Ana Paula Silva Caldas, Ana Cristina Simões e Silva, Josefina Bressan, Helen Hermana Miranda Hermsdorff, (2022) Nut enriched energy restricted diet has potential to decrease hunger in women at cardiometabolic risk: a randomized controlled trial (Brazilian Nuts Study), Nutrition Research, doi: https://doi.org/10.1016/j.nutres.2022.11.003

Supplementary Table S5. JBI CRITICAL APPRAISAL CHECKLIST FOR RANDOMIZED CONTROLLED TRIALS INDIVIDUAL

Reviewer: consensus between both reviewers____________
Date____________________________

Author_Wien______________________________________Year 2003 Record Number________

	Yes	No	Unclear	NA
1. Was true randomization used for assignment of participants to treatment groups?	X	☐	☐	☐
2. Was allocation to treatment groups concealed?	X	☐	☐	☐
3. Were treatment groups similar at the baseline?	X	☐	☐	☐
4. Were participants blind to treatment assignment?	☐	X	☐	☐
5. Were those delivering treatment blind to treatment assignment?	☐	X	☐	☐
6. Were outcomes assessors blind to treatment assignment?		☐	X	☐
7. Were treatment groups treated identically other than the intervention of interest?	X	☐	☐	☐
8. Was follow up complete and if not, were differences between groups in terms of their follow up adequately described and analyzed?	X	☐	☐	☐
9. Were participants analyzed in the groups to which they were randomized?	X	☐	☐	☐
10. Were outcomes measured in the same way for treatment groups?	X	☐	☐	☐
11. Were outcomes measured in a reliable way?	☐	☐	X	☐
12. Was appropriate statistical analysis used?	X	☐	☐	☐
13. Was the trial design appropriate, and any deviations from the standard RCT design (individual randomization, parallel groups) accounted for in the conduct and analysis of the trial?	☐	☐	☐	☐

Overall appraisal: Include ☐ Exclude ☐ Seek further info ☐

Reviewer consensus between both reviewers Date____________________________

Author_LI_________________________________Year__2010_ Record Number________

	Yes	No	Unclear	NA
1. Was true randomization used for assignment of participants to treatment groups?	X	☐	☐	☐
2. Was allocation to treatment groups concealed?	X	☐	☐	☐
3. Were treatment groups similar at the baseline?	X	☐	☐	☐
4. Were participants blind to treatment assignment?		X	☐	☐
5. Were those delivering treatment blind to treatment assignment?	☐	X	☐	☐
6. Were outcomes assessors blind to treatment assignment?	☐	☐	X	☐
7. Were treatment groups treated identically other than the intervention of interest?	X	☐	☐	☐
8. Was follow up complete and if not, were differences between groups in terms of their follow up adequately described and analyzed?	X	☐	☐	☐
9. Were participants analyzed in the groups to which they were randomized?	☐	X	☐	☐
10. Were outcomes measured in the same way for treatment groups?	X	☐	☐	☐
11. Were outcomes measured in a reliable way?	☐	☐	X	☐
12. Was appropriate statistical analysis used?	X	☐	☐	☐
13. Was the trial design appropriate, and any deviations from the standard RCT design (individual randomization, parallel groups) accounted for in the conduct and analysis of the trial?	☐	☐	☐	☐

Overall appraisal: Include ☐ Exclude ☐ Seek further info ☐

JBI CRITICAL APPRAISAL CHECKLIST FOR RANDOMIZED CONTROLLED TRIALS

Reviewer consensus between both reviewers Date______________________________

Author______FOSTER_________________________________ __Year____2012_____ Record Number_________

	Yes	No	Unclear	NA
1. Was true randomization used for assignment of participants to treatment groups?	X	☐	☐	☐
2. Was allocation to treatment groups concealed?	X	☐	☐	☐
3. Were treatment groups similar at the baseline?	X	☐	☐	☐
4. Were participants blind to treatment assignment?	☐	X	☐	☐
5. Were those delivering treatment blind to treatment assignment?	☐	X	☐	☐
6. Were outcomes assessors blind to treatment assignment?	☐	☐	X	☐
7. Were treatment groups treated identically other than the intervention of interest?	X	☐	☐	☐
8. Was follow up complete and if not, were differences between groups in terms of their follow up adequately described and analyzed?	X	☐	☐	☐
9. Were participants analyzed in the groups to which they were randomized?	X	☐	☐	☐
10. Were outcomes measured in the same way for treatment groups?	X	☐	☐	☐
11. Were outcomes measured in a reliable way?	☐	☐	X	☐
12. Was appropriate statistical analysis used?	X	☐	☐	☐
13. Was the trial design appropriate, and any deviations from the standard RCT design (individual randomization, parallel groups) accounted for in the conduct and analysis of the trial?	X	☐	☐	☐

Overall appraisal: Include ☐ Exclude ☐ Seek further info ☐

JBI CRITICAL APPRAISAL CHECKLIST FOR RANDOMIZED CONTROLLED TRIALS

Reviewer consensus between both reviewers Date_______________________________

Author___Abazarfard____________________________Year__2013_______Record Number________

	Yes	No	Unclear	NA
1. Was true randomization used for assignment of participants to treatment groups?	X	☐	☐	☐
2. Was allocation to treatment groups concealed?	X	☐	☐	☐
3. Were treatment groups similar at the baseline?	X	☐	☐	☐
4. Were participants blind to treatment assignment?	☐	X	☐	☐
5. Were those delivering treatment blind to treatment assignment?	☐	X	☐	☐
6. Were outcomes assessors blind to treatment assignment?	☐	☐	X	☐
7. Were treatment groups treated identically other than the intervention of interest?	X	☐	☐	☐
8. Was follow up complete and if not, were differences between groups in terms of their follow up adequately described and analyzed?	X	☐	☐	☐
9. Were participants analyzed in the groups to which they were randomized?	☐	X	☐	☐
10. Were outcomes measured in the same way for treatment groups?	X	☐	☐	☐
11. Were outcomes measured in a reliable way?	☐	☐	X	☐
12. Was appropriate statistical analysis used?	X	☐	☐	☐
13. Was the trial design appropriate, and any deviations from the standard RCT design (individual randomization, parallel groups) accounted for in the conduct and analysis of the trial?	☐	☐	☐	☐

Overall appraisal: Include ☐ Exclude ☐ Seek further info ☐

JBI CRITICAL APPRAISAL CHECKLIST FOR RANDOMIZED CONTROLLED TRIALS

Reviewer consensus between both reviewers Date______________________________

Author_Alves ________________________________ Year_________ Record Number_________

	Yes	No	Unclear	NA
1. Was true randomization used for assignment of participants to treatment groups?	☐	☐	X	☐
2. Was allocation to treatment groups concealed?	☐	☐	X	☐
3. Were treatment groups similar at the baseline?	X	☐	☐	☐
4. Were participants blind to treatment assignment?	☐	X	☐	☐
5. Were those delivering treatment blind to treatment assignment?	☐	X	☐	☐
6. Were outcomes assessors blind to treatment assignment?	☐	☐	X	☐
7. Were treatment groups treated identically other than the intervention of interest?	X	☐	☐	☐
8. Was follow up complete and if not, were differences between groups in terms of their follow up adequately described and analyzed?	X	☐	☐	☐
9. Were participants analyzed in the groups to which they were randomized?	☐	X	☐	☐
10. Were outcomes measured in the same way for treatment groups?	X	☐	☐	☐
11. Were outcomes measured in a reliable way?	☐	☐	X	☐
12. Was appropriate statistical analysis used?	X	☐	☐	☐
13. Was the trial design appropriate, and any deviations from the standard RCT design (individual randomization, parallel groups) accounted for in the conduct and analysis of the trial?	X	☐	☐	☐

Overall appraisal: Include ☐ Exclude ☐ Seek further info ☐

Comments (Including reason for exclusion)

JBI CRITICAL APPRAISAL CHECKLIST FOR RANDOMIZED CONTROLLED TRIALS

Reviewer consensus between both reviewers Date______________________________

Author Abazarfard________________________________ Year__2016_______ Record Number_________

	Yes	No	Unclear	NA
1. Was true randomization used for assignment of participants to treatment groups?	X	☐	☐	☐
2. Was allocation to treatment groups concealed?	X	☐	☐	☐
3. Were treatment groups similar at the baseline?	X	☐	☐	☐
4. Were participants blind to treatment assignment?	☐	X	☐	☐
5. Were those delivering treatment blind to treatment assignment?	☐	X	☐	☐
6. Were outcomes assessors blind to treatment assignment?	☐	☐	X	☐
7. Were treatment groups treated identically other than the intervention of interest?	X	☐	☐	☐
8. Was follow up complete and if not, were differences between groups in terms of their follow up adequately described and analyzed?	X	☐	☐	☐
9. Were participants analyzed in the groups to which they were randomized?	☐	X	☐	☐
10. Were outcomes measured in the same way for treatment groups?	X	☐	☐	☐
11. Were outcomes measured in a reliable way?	☐	☐	X	☐
12. Was appropriate statistical analysis used?	X	☐	☐	☐
13. Was the trial design appropriate, and any deviations from the standard RCT design (individual randomization, parallel groups) accounted for in the conduct and analysis of the trial?	X	☐	☐	☐

Overall appraisal: Include ☐ Exclude ☐ Seek further info ☐

Comments (Including reason for exclusion)

JBI CRITICAL APPRAISAL CHECKLIST FOR RANDOMIZED CONTROLLED TRIALS

Reviewer____
Date______________________________

Author_Dhillon______________________________________Year_2016_____ Record Number_________

		Yes	No	Unclear	NA
1.	Was true randomization used for assignment of participants to treatment groups?	☐	☐	X	☐
2.	Was allocation to treatment groups concealed?	☐	☐	X	☐
3.	Were treatment groups similar at the baseline?	X	☐	☐	☐
4.	Were participants blind to treatment assignment?	☐	X	☐	☐
5.	Were those delivering treatment blind to treatment assignment?	☐	X	☐	☐
6.	Were outcomes assessors blind to treatment assignment?	☐	☐	X	☐
7.	Were treatment groups treated identically other than the intervention of interest?	X	☐	☐	☐
8.	Was follow up complete and if not, were differences between groups in terms of their follow up adequately described and analyzed?	X	☐	☐	☐
9.	Were participants analyzed in the groups to which they were randomized?	X	☐	☐	☐
10.	Were outcomes measured in the same way for treatment groups?	X	☐	☐	☐
11.	Were outcomes measured in a reliable way?	☐	☐	X	☐
12.	Was appropriate statistical analysis used?	X	☐	☐	☐
13.	Was the trial design appropriate, and any deviations from the standard RCT design (individual randomization, parallel groups) accounted for in the conduct and analysis of the trial?	X	☐	☐	☐

Overall appraisal: Include ☐ Exclude ☐ Seek further info ☐

JBI CRITICAL APPRAISAL CHECKLIST FOR RANDOMIZED CONTROLLED TRIALS

Reviewer consensus between both reviewers Date________________________________

Author___Rock_____________________________________ _ _ _ _ _Year__2017__ Record Number_________

	Yes	No	Unclear	NA
1. Was true randomization used for assignment of participants to treatment groups?	☐	☐	X	☐
2. Was allocation to treatment groups concealed?	☐	☐	X	☐
3. Were treatment groups similar at the baseline?	X	☐	☐	☐
4. Were participants blind to treatment assignment?	☐	X	☐	☐
5. Were those delivering treatment blind to treatment assignment?	☐	X	☐	☐
6. Were outcomes assessors blind to treatment assignment?	☐	☐	X	☐
7. Were treatment groups treated identically other than the intervention of interest?	X	☐	☐	☐
8. Was follow up complete and if not, were differences between groups in terms of their follow up adequately described and analyzed?	☐	X	☐	☐
9. Were participants analyzed in the groups to which they were randomized?	☐	X	☐	☐
10. Were outcomes measured in the same way for treatment groups?	X	☐	☐	☐
11. Were outcomes measured in a reliable way?	☐	☐	X	☐
12. Was appropriate statistical analysis used?	X	☐	☐	☐
13. Was the trial design appropriate, and any deviations from the standard RCT design (individual randomization, parallel groups) accounted for in the conduct and analysis of the trial?	X	☐	☐	☐

Overall appraisal: Include ☐ Exclude ☐ Seek further info ☐

JBI CRITICAL APPRAISAL CHECKLIST FOR RANDOMIZED CONTROLLED TRIALS

Reviewer____ ___________________________________

Date______________________________

Author___Fatahi______________________________________Year________ Record Number________

	Yes	No	Unclear	NA
1. Was true randomization used for assignment of participants to treatment groups?	X	☐	☐	☐
2. Was allocation to treatment groups concealed?	X	☐	☐	☐
3. Were treatment groups similar at the baseline?	X	☐	☐	☐
4. Were participants blind to treatment assignment?	☐	X	☐	☐
5. Were those delivering treatment blind to treatment assignment?	☐	X	☐	☐
6. Were outcomes assessors blind to treatment assignment?	☐	☐	X	☐
7. Were treatment groups treated identically other than the intervention of interest?	X	☐	☐	☐
8. Was follow up complete and if not, were differences between groups in terms of their follow up adequately described and analyzed?	X	☐	☐	☐
9. Were participants analyzed in the groups to which they were randomized?	X	☐	☐	☐
10. Were outcomes measured in the same way for treatment groups?	X	☐	☐	☐
11. Were outcomes measured in a reliable way?	X	☐	☐	☐
12. Was appropriate statistical analysis used?	X	☐	☐	☐
13. Was the trial design appropriate, and any deviations from the standard RCT design (individual randomization, parallel groups) accounted for in the conduct and analysis of the trial?	X	☐	☐	☐

Overall appraisal: Include ☐ Exclude ☐ Seek further info ☐

JBI CRITICAL APPRAISAL CHECKLIST FOR RANDOMIZED CONTROLLED TRIALS

Reviewer____
Date______________________________

Author___Rock____Year__2020__ Record Number_________

	Yes	No	Unclear	NA
1. Was true randomization used for assignment of participants to treatment groups?	X	☐	☐	☐
2. Was allocation to treatment groups concealed?	☐	☐	X	☐
3. Were treatment groups similar at the baseline?	X	☐	☐	☐
4. Were participants blind to treatment assignment?	☐	X	☐	☐
5. Were those delivering treatment blind to treatment assignment?	☐	X	☐	☐
6. Were outcomes assessors blind to treatment assignment?	☐	☐	X	☐
7. Were treatment groups treated identically other than the intervention of interest?	X	☐	☐	☐
8. Was follow up complete and if not, were differences between groups in terms of their follow up adequately described and analyzed?	☐	X	☐	☐
9. Were participants analyzed in the groups to which they were randomized?	☐	X	☐	☐
10. Were outcomes measured in the same way for treatment groups?	X	☐	☐	☐
11. Were outcomes measured in a reliable way?	☐	☐	X	☐
12. Was appropriate statistical analysis used?	X	☐	☐	☐
13. Was the trial design appropriate, and any deviations from the standard RCT design (individual randomization, parallel groups) accounted for in the conduct and analysis of the trial?	X	☐	☐	☐

Overall appraisal: Include ☐ Exclude ☐ Seek further info ☐

JBI CRITICAL APPRAISAL CHECKLIST FOR RANDOMIZED CONTROLLED TRIALS

Reviewer____
Date______________________________

Author__Fialho___________________________________Year__2021_____ Record Number_________

	Yes	No	Unclear	NA
1. Was true randomization used for assignment of participants to treatment groups?	X	☐	☐	☐
2. Was allocation to treatment groups concealed?	☐	☐	X	☐
3. Were treatment groups similar at the baseline?	X	☐	☐	☐
4. Were participants blind to treatment assignment?	☐	X	☐	☐
5. Were those delivering treatment blind to treatment assignment?	☐	X	☐	☐
6. Were outcomes assessors blind to treatment assignment?	☐	☐	X	☐
7. Were treatment groups treated identically other than the intervention of interest?	X	☐	☐	☐
8. Was follow up complete and if not, were differences between groups in terms of their follow up adequately described and analyzed?	X	☐	☐	☐
9. Were participants analyzed in the groups to which they were randomized?	☐	X	☐	☐
10. Were outcomes measured in the same way for treatment groups?	X	☐	☐	☐
11. Were outcomes measured in a reliable way?	☐	☐	X	☐
12. Was appropriate statistical analysis used?	X	☐	☐	☐
13. Was the trial design appropriate, and any deviations from the standard RCT design (individual randomization, parallel groups) accounted for in the conduct and analysis of the trial?	X	☐	☐	☐

Overall appraisal: Include ☐ Exclude ☐ Seek further info ☐

JBI CRITICAL APPRAISAL CHECKLIST FOR RANDOMIZED CONTROLLED TRIALS

Reviewer____

Date______________________________

Author__Ghanavati,Parsa,Nasrollahzadeh________________Year__2021_____Record Number________

	Yes	No	Unclear	NA
1. Was true randomization used for assignment of participants to treatment groups?	X	☐	☐	☐
2. Was allocation to treatment groups concealed?	☐	☐	X	☐
3. Were treatment groups similar at the baseline?	X	☐	☐	☐
4. Were participants blind to treatment assignment?	☐	X	☐	☐
5. Were those delivering treatment blind to treatment assignment?	☐	X	☐	☐
6. Were outcomes assessors blind to treatment assignment?	☐	☐	X	☐
7. Were treatment groups treated identically other than the intervention of interest?	X	☐	☐	☐
8. Was follow up complete and if not, were differences between groups in terms of their follow up adequately described and analyzed?	☐	X	☐	☐
9. Were participants analyzed in the groups to which they were randomized?	☐	X	☐	☐
10. Were outcomes measured in the same way for treatment groups?	X	☐	☐	☐
11. Were outcomes measured in a reliable way?	☐	☐	X	☐
12. Was appropriate statistical analysis used?	X	☐	☐	☐

13. Was the trial design appropriate, and any deviations from the standard RCT design (individual randomization, parallel groups) accounted for in the conduct and analysis of the trial? X ☐ ☐ ☐

Overall appraisal: Include ☐ Exclude ☐ Seek further info ☐

JBI CRITICAL APPRAISAL CHECKLIST FOR RANDOMIZED CONTROLLED TRIALS

Reviewer____

Date________________________________

Author__Ghanavati__et al.,__________________Year__2021_______ Record Number_________

	Yes	No	Unclear	NA
1. Was true randomization used for assignment of participants to treatment groups?	X	☐	☐	☐
2. Was allocation to treatment groups concealed?	☐	☐	X	☐
3. Were treatment groups similar at the baseline?	X	☐	☐	☐
4. Were participants blind to treatment assignment?	☐	X	☐	☐
5. Were those delivering treatment blind to treatment assignment?	☐	X	☐	☐
6. Were outcomes assessors blind to treatment assignment?	☐	☐	X	☐
7. Were treatment groups treated identically other than the intervention of interest?	X	☐	☐	☐
8. Was follow up complete and if not, were differences between groups in terms of their follow up adequately described and analyzed?	X	☐	☐	☐
9. Were participants analyzed in the groups to which they were randomized?	☐	X	☐	☐
10. Were outcomes measured in the same way for treatment groups?	X	☐	☐	☐
11. Were outcomes measured in a reliable way?	☐	☐	X	☐
12. Was appropriate statistical analysis used?	X	☐	☐	☐
13. Was the trial design appropriate, and any deviations from the standard RCT design (individual randomization, parallel groups) accounted for in the conduct and analysis of the trial?	X	☐	☐	☐

Overall appraisal: Include ☐ Exclude ☐ Seek further info ☐

JBI CRITICAL APPRAISAL CHECKLIST FOR RANDOMIZED CONTROLLED TRIALS

Reviewer____
Date_________________________________

Author_____Caldas_____________________________Year 2022____ Record Number_________

	Yes	No	Unclea r	NA
1. Was true randomization used for assignment of participants to treatment groups?	☐	☐	X	☐
2. Was allocation to treatment groups concealed?	☐	☐	X	☐
3. Were treatment groups similar at the baseline?	X	☐	☐	☐
4. Were participants blind to treatment assignment?	☐	X	☐	☐
5. Were those delivering treatment blind to treatment assignment?	☐	X	☐	☐
6. Were outcomes assessors blind to treatment assignment?	☐	☐	X	☐
7. Were treatment groups treated identically other than the intervention of interest?	X	☐	☐	☐
8. Was follow up complete and if not, were differences between groups in terms of their follow up adequately described and analyzed?	X	☐	☐	☐
9. Were participants analyzed in the groups to which they were randomized?	☐	X	☐	☐
10. Were outcomes measured in the same way for treatment groups?	X	☐	☐	☐
11. Were outcomes measured in a reliable way?	☐	☐	X	☐
12. Was appropriate statistical analysis used?	X	☐	☐	☐
13. Was the trial design appropriate, and any deviations from the standard RCT design (individual randomization, parallel groups) accounted for in the conduct and analysis of the trial?	X	☐	☐	☐

Overall appraisal: Include ☐ Exclude ☐ Seek further info ☐

JBI CRITICAL APPRAISAL CHECKLIST FOR RANDOMIZED CONTROLLED TRIALS

Reviewer Reviewer consensus between both reviewers Date______________________________

Author_Petersen_______________________________ _ _ _ _Year___2022______ Record Number_________

	Yes	No	Unclear	NA
1. Was true randomization used for assignment of participants to treatment groups?	X	□	□	□
2. Was allocation to treatment groups concealed?	X	□	□	□
3. Were treatment groups similar at the baseline?	X	□	□	□
4. Were participants blind to treatment assignment?	□	X	□	□
5. Were those delivering treatment blind to treatment assignment?	□	X	□	□
6. Were outcomes assessors blind to treatment assignment?	□	□	X	□
7. Were treatment groups treated identically other than the intervention of interest?	X	□	□	□
8. Was follow up complete and if not, were differences between groups in terms of their follow up adequately described and analyzed?	□	X	□	□
9. Were participants analyzed in the groups to which they were randomized?	X	□	□	□
10. Were outcomes measured in the same way for treatment groups?	X	□	□	□
11. Were outcomes measured in a reliable way?	□	□	X	□
12. Was appropriate statistical analysis used?	X	□	□	□
13. Was the trial design appropriate, and any deviations from the standard RCT design (individual randomization, parallel groups) accounted for in the conduct and analysis of the trial?	X	□	□	□

Overall appraisal: Include ☐ Exclude ☐ Seek further info ☐

JBI CRITICAL APPRAISAL CHECKLIST FOR RANDOMIZED CONTROLLED TRIALS

Reviewer Reviewer consensus between both reviewers Date______________________________

Author___Rocha__________________________Year__2022______ Record Number_________

	Yes	No	Unclear	NA
1. Was true randomization used for assignment of participants to treatment groups?	X	☐	☐	☐
2. Was allocation to treatment groups concealed?	X	☐	☐	☐
3. Were treatment groups similar at the baseline?	X	☐	☐	☐
4. Were participants blind to treatment assignment?	☐	X	☐	☐
5. Were those delivering treatment blind to treatment assignment?	☐	X	☐	☐
6. Were outcomes assessors blind to treatment assignment?	X	☐	☐	☐
7. Were treatment groups treated identically other than the intervention of interest?	X	☐	☐	☐
8. Was follow up complete and if not, were differences between groups in terms of their follow up adequately described and analyzed?	X	☐	☐	☐
9. Were participants analyzed in the groups to which they were randomized?	☐	X	☐	☐
10. Were outcomes measured in the same way for treatment groups?	X	☐	☐	☐
11. Were outcomes measured in a reliable way?	☐	☐	X	☐
12. Was appropriate statistical analysis used?	X	☐	☐	☐

	Yes	No	Unclear	NA
13. Was the trial design appropriate, and any deviations from the standard RCT design (individual randomization, parallel groups) accounted for in the conduct and analysis of the trial?	X	☐	☐	☐

Overall appraisal: Include ☐ Exclude ☐ Seek further info ☐

JBI CRITICAL APPRAISAL CHECKLIST FOR RANDOMIZED CONTROLLED TRIALS

Reviewer Reviewer consensus between both reviewers Date______________________________

Author_Petersen______________________________-----Year___2022______ Record Number_________

	Yes	No	Unclear	NA
1. Was true randomization used for assignment of participants to treatment groups?	X	☐	☐	☐
2. Was allocation to treatment groups concealed?	X	☐	☐	☐
3. Were treatment groups similar at the baseline?	X	☐	☐	☐
4. Were participants blind to treatment assignment?	☐	X	☐	☐
5. Were those delivering treatment blind to treatment assignment?	☐	X	☐	☐
6. Were outcomes assessors blind to treatment assignment?	☐	☐	X	☐
7. Were treatment groups treated identically other than the intervention of interest?	X	☐	☐	☐
8. Was follow up complete and if not, were differences between groups in terms of their follow up adequately described and analyzed?	☐	X	☐	☐
9. Were participants analyzed in the groups to which they were randomized?	X	☐	☐	☐
10. Were outcomes measured in the same way for treatment groups?	X	☐	☐	☐
11. Were outcomes measured in a reliable way?	☐	☐	X	☐
12. Was appropriate statistical analysis used?	X	☐	☐	☐

13. Was the trial design appropriate, and any deviations from the standard RCT design (individual randomization, parallel groups) accounted for in the conduct and analysis of the trial? X ☐ ☐ ☐

Overall appraisal: Include ☐ Exclude ☐ Seek further info ☐

Supplementary Table S6. Transitivity assessment regarding the intervention group

Reference	Intervention	n	Baseline age	Baseline BW	Baseline BMI	Baseline WC	Baseline BF%
**Wien et al., 2003**	Almonds	32	55	113	38	122	42
**Li et al 2010**	Pistachio	36	45.4	86	30.1	-	-
**Foster et al 2012**	Almonds	61	47.0	94.0	33.9	-	-
**Abazarfard et al., 2014**	Almonds	50	42.36	76.39	29.91	107.78	-
**Alves et al., 2014 peanut CVP**	Peanuts	22	28	93.4	29.5	100.9	31.1
**Alves et al., 2014 peanut HOP**	Peanuts	21	26.8	95.1	29.9	101.7	33.5
**Abazarfard et al 2016**	Almonds	54	42.36	76.39	29.91	107.78	-
**Rock et al., 2017**	Walnut	49	53.3	91.1	32.4	111.5	-
**Dhillon, Tan, Mattes 2016**	Almonds	43	31.1	82.8	29.9	88.1	-
**Caldas et al., 2019 CVP**	Peanut	24	28	92.7	29,4	100.5	
**Caldas et al., 2019 HOP**	Peanut	27	27.2	95.5	30.1	102.3	-
**Fatahi et al., 2019**	Walnut	33	54.01	85.6	108.0	108.0	-
**Rock et al., 2020**	Pistachio	50	55.0	94.7	32.8	108.4	-
**Fialho et al., 2021 WP**	Peanut	8	33.1	84.2	32.3	98.9	38.0

**Ghanavati, Parsa, Nasrollahzadeh, 2021**	Mixed nuts equal amounts of unsalted pistachios, almonds and peanuts	35	58	80.5	30.0	-	33.0
**Ghanavati et al., 2021**	Mixed nuts equal amounts of unsalted pistachios, almonds and peanuts	35	58.23	79.4	29.8	105.0	-
**Caldas et al., 2022**	15 g of Brazil nuts + 30 g of cashew nuts	14	31.2	90·5	33·8	107·7	48·7
**Petersen et al., 2022**	Peanut	50	59	91.6	33.1	109	-

Supplementary Table S7. Transitivity assessment regarding the intervention groups

Reference	control	n	Baseline age	Baseline BW	Baseline BMI	Baseline WC	Baseline BF%
**Wien et al., 2003**	Control	32	57	114	37	117	43
**Li et al 2010**	Control	36	47.3	85.5	30.9	-	-
**Foster et al 2012**	Control	62	46.7	91.5	34.0	-	-
**Abazarfard et al., 2014**	Control	50	42.94	75.58	29.37	106.20	-
**Alves et al., 2014**	Control	22	27.4	94.5	29.7	102.3	33.4
**Abazarfard et al 2016**	Control	50	42.94	75.58	29.37	106.20	-
**Dhillon, Tan, Mattes 2016**	Control	43	31	84.7	40	90.2	-
**Rock et al., 2017**	Control	51	52.2	90.9	32.4	109.9	
**Caldas et al., 2019**	Control	22	27.4	94·5	29·7	102·2	-
**Fatahi et al., 2019**	Control	33	52.9	83.9	-	109.0	-
**Rock et al., 2020**	Control	50	56.2	93.8	32.8	108.6	-
**Fialho et al., 2021**	Control	8	33.1	83.7	32.8	95.7	38.4
**Ghanavati, Parsa, Nasrollahzadeh, 2021**	Control	32	59	84.8	31.8	-	34.2
**Ghanavati et al., 2021**	Control	32	58.86	83.7	31.5	107.4	-
**Caldas et al., 2022**	Control	15	31·6	87.9	33.0	107·7	48·08
**Petersen et al., 2022**	Control	50	58	92.9	33.0	109	-
**Rocha et al., 2022**	Control	14	32.2	89.3	33.4	108.9	44.5

A

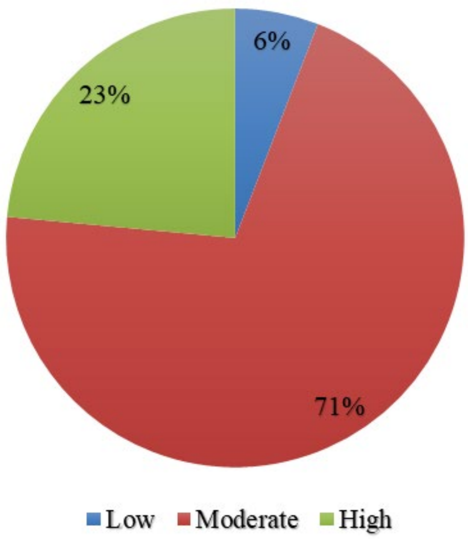

B

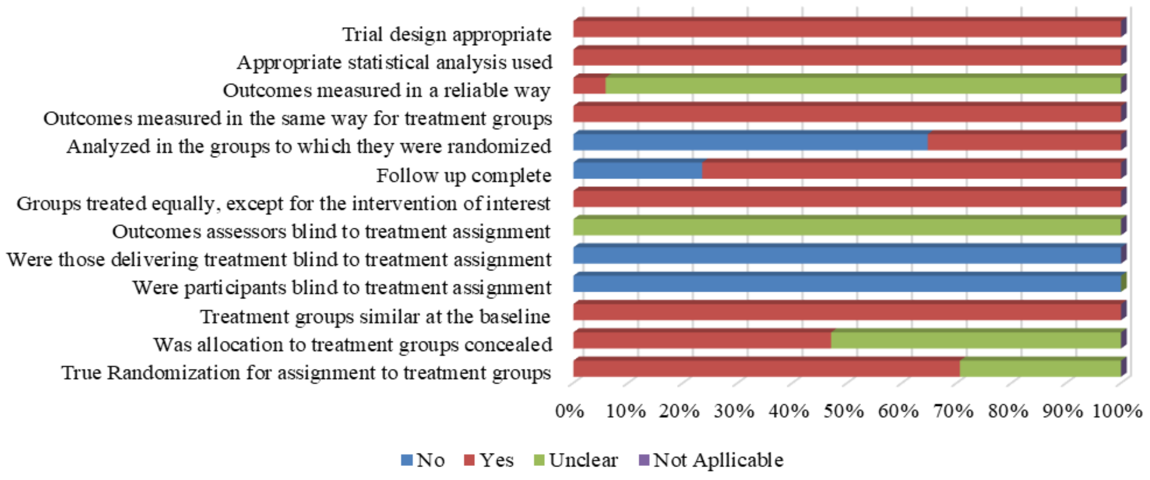

Figure S1. Risk of bias for each study evaluated and summary of responses presented as percentages in all randomized clinical trials included in the systematic review.

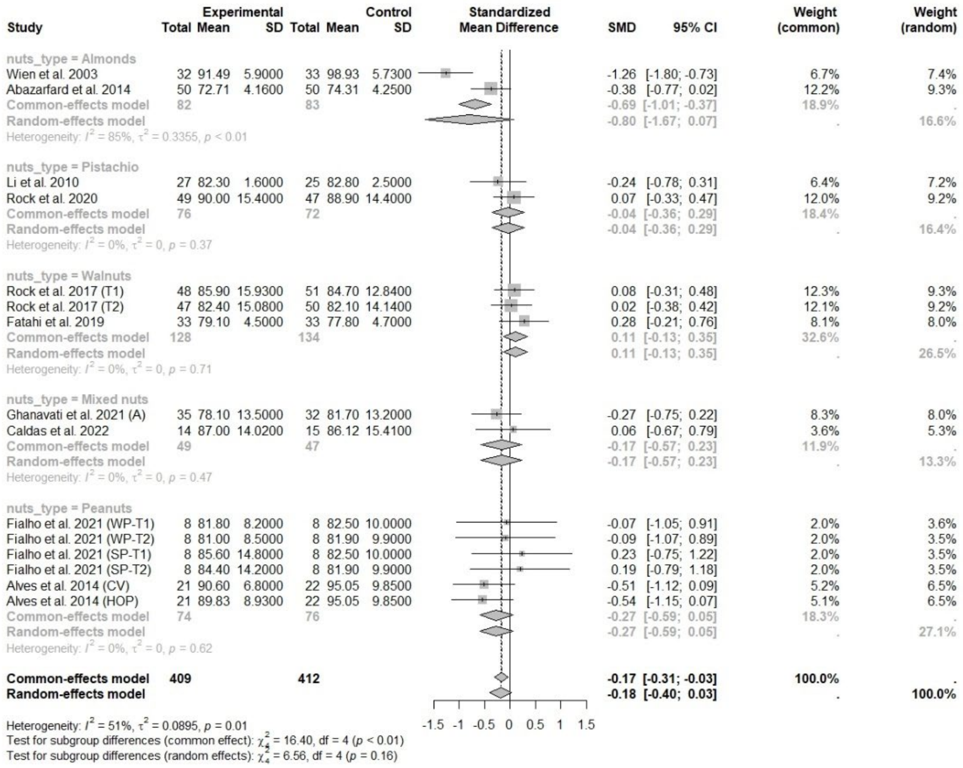

Supplementary Figure S2 Forest plot of randomized controlled trials investigating the effects of nuts consumption combined with energy-restricted diet on weight according to nuts type. Squares represent the weight of studies in meta-analysis and horizontal lines their 95 % CI; diamond’s center represents the combined treatment effect and horizontal tips represent the 95 % CI. CI: confidence intervals; SMD: standard mean differences.

T1, first assessment after intervention; T2, second assessment after intervention; CV, conventional peanuts group; HOP, high-oleic peanuts group; WP, whole roasted peanuts group; SP, skinned peanuts group.

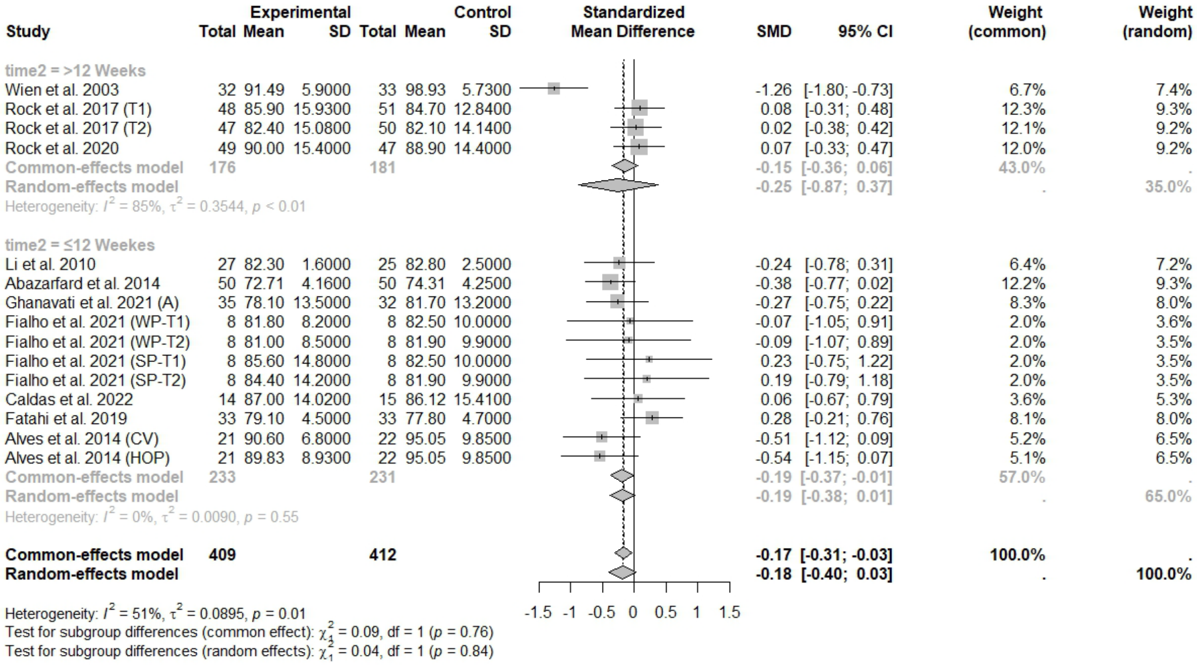

Supplementary Figure S3. Forest plot of randomized controlled trials investigating the effects of nuts consumption combined with to energy-restricted diet on weight according to time of intervention (≤12 and >12 weeks). Squares represent the weight of studies in meta-analysis and horizontal lines their 95 % CI; diamond's center represents the combined treatment effect and horizontal tips represent the 95 % CI. CI: confidence intervals; SMD: standard mean differences.

T1, first assessment after intervention; T2, second assessment after intervention; CV, conventional peanuts group; HOP, high-oleic peanuts group; WP, whole roasted peanuts group; SP, skinned peanuts group.

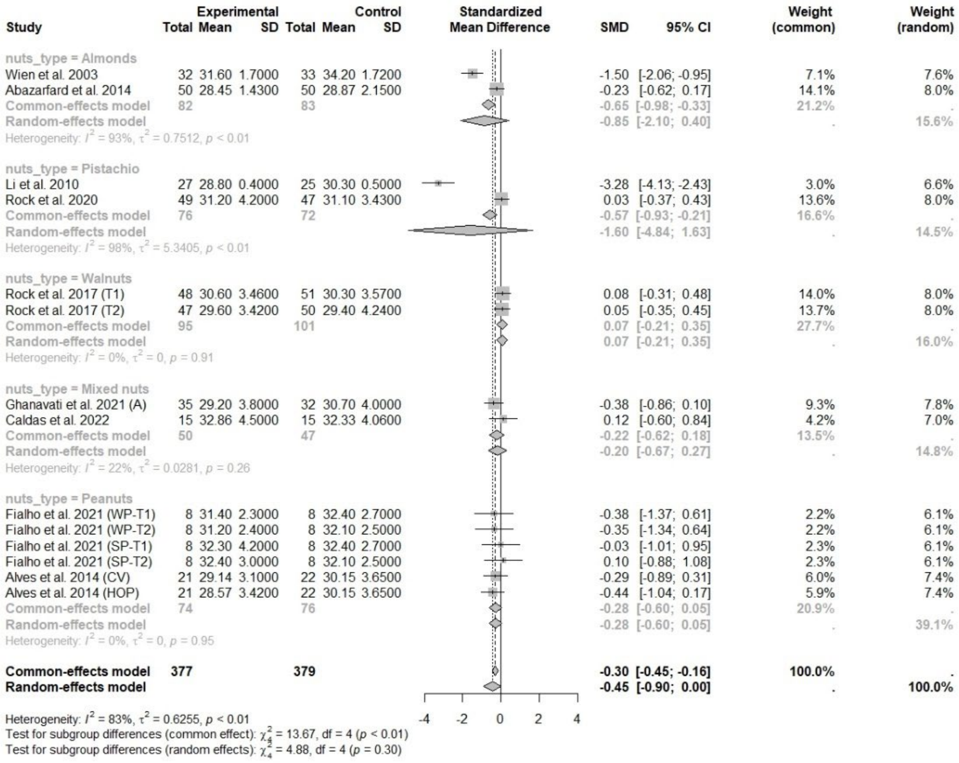

Supplementary Figure S4. Forest plot of randomized controlled trials investigating the effects of nuts consumption combined with energy-restricted diet on body mass index according to nuts type. Squares represent the weight of studies in meta-analysis and horizontal lines their 95 % CI; diamond's center represents the combined treatment effect and horizontal tips represent the 95 % CI. CI: confidence intervals; SMD: standard mean differences.

T1, first assessment after intervention; T2, second assessment after intervention; CV, conventional peanuts group; HOP, high-oleic peanuts group; WP, whole roasted peanuts group; SP, skinned peanuts group.

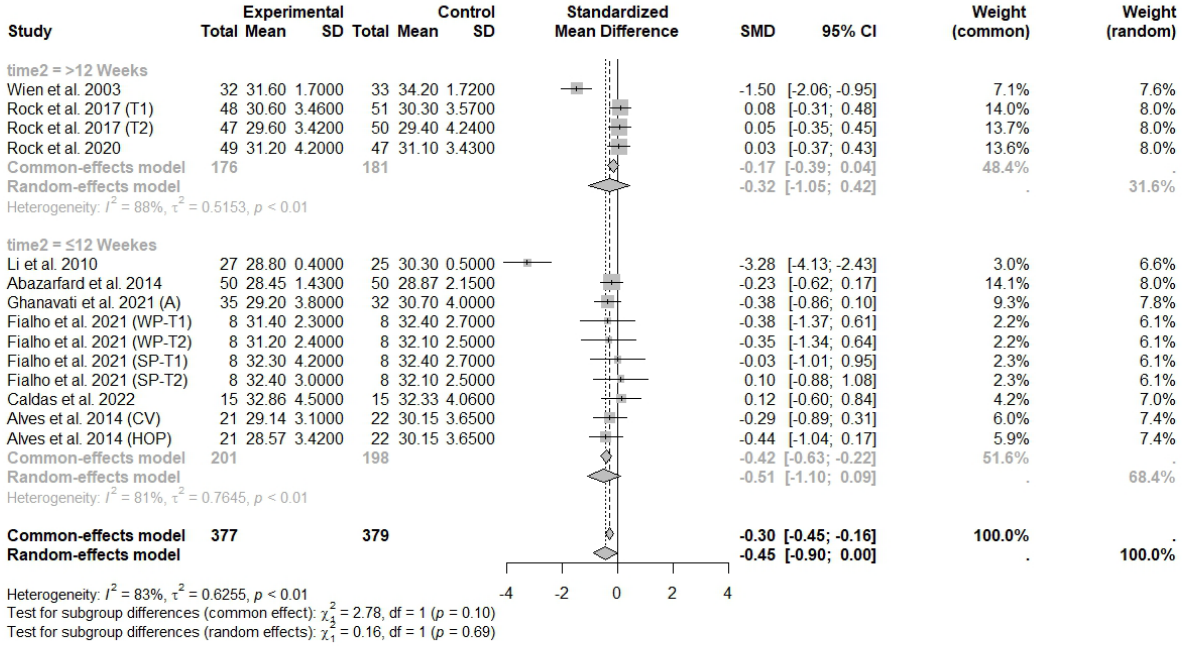

Supplementary Figure S5. Forest plot of randomized controlled trials investigating the effects of nuts consumption combined with energy-restricted diet on body mass index according to time of intervention (≤12 and >12 weeks). Squares represent the weight of studies in meta-analysis and horizontal lines their 95 % CI; diamond's center represents the combined treatment effect and horizontal tips represent the 95 % CI. CI: confidence intervals; SMD: standard mean differences.

T1, first assessment after intervention; T2, second assessment after intervention; CV, conventional peanuts group; HOP, high-oleic peanuts group; WP, whole roasted peanuts group; SP, skinned peanuts group.

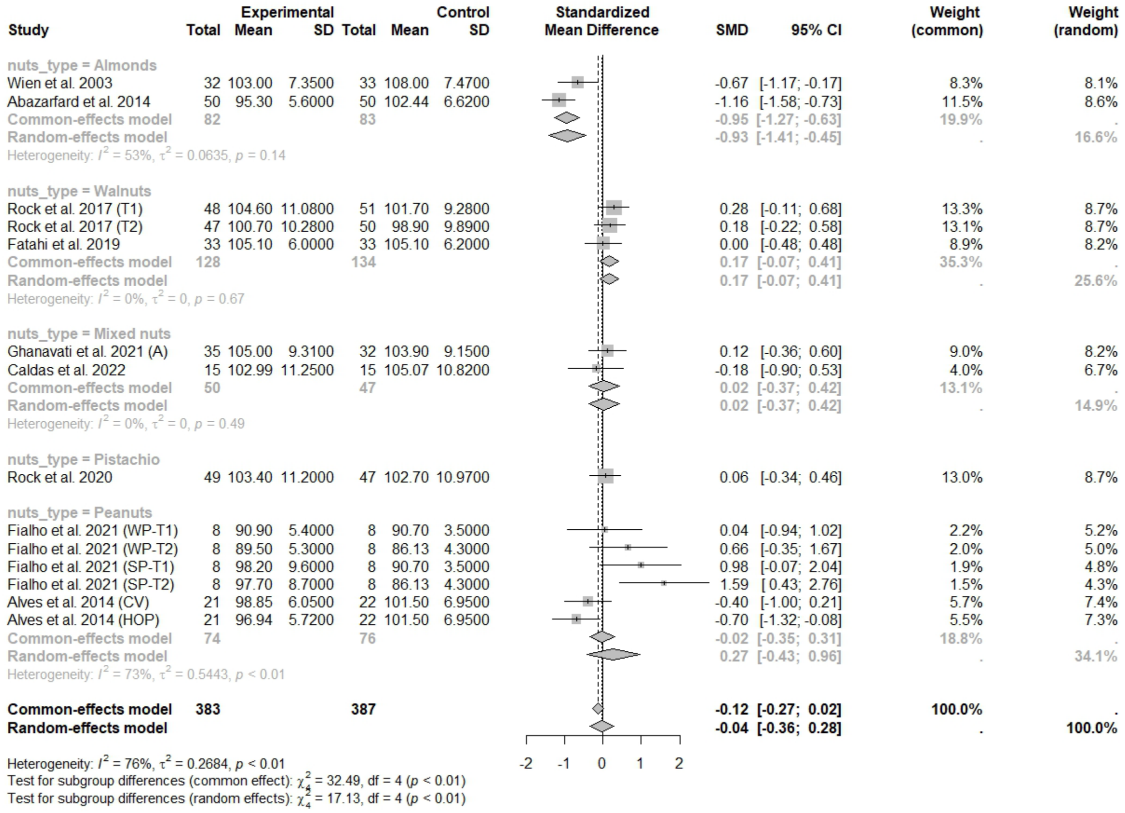

Supplementary Figure S6. Forest plot of randomized controlled trials investigating the effects of nuts consumption combined with energy-restricted diet on waist circumference according to nuts type. Squares represent the weight of studies in meta-analysis and horizontal lines their 95 % CI; diamond's center represents the combined treatment effect and horizontal tips represent the 95 % CI. CI: confidence intervals; SMD: standard mean differences.

T1, first assessment after intervention; T2, second assessment after intervention; CV, conventional peanuts group; HOP, high-oleic peanuts group; WP, whole roasted peanuts group; SP, skinned peanuts group.

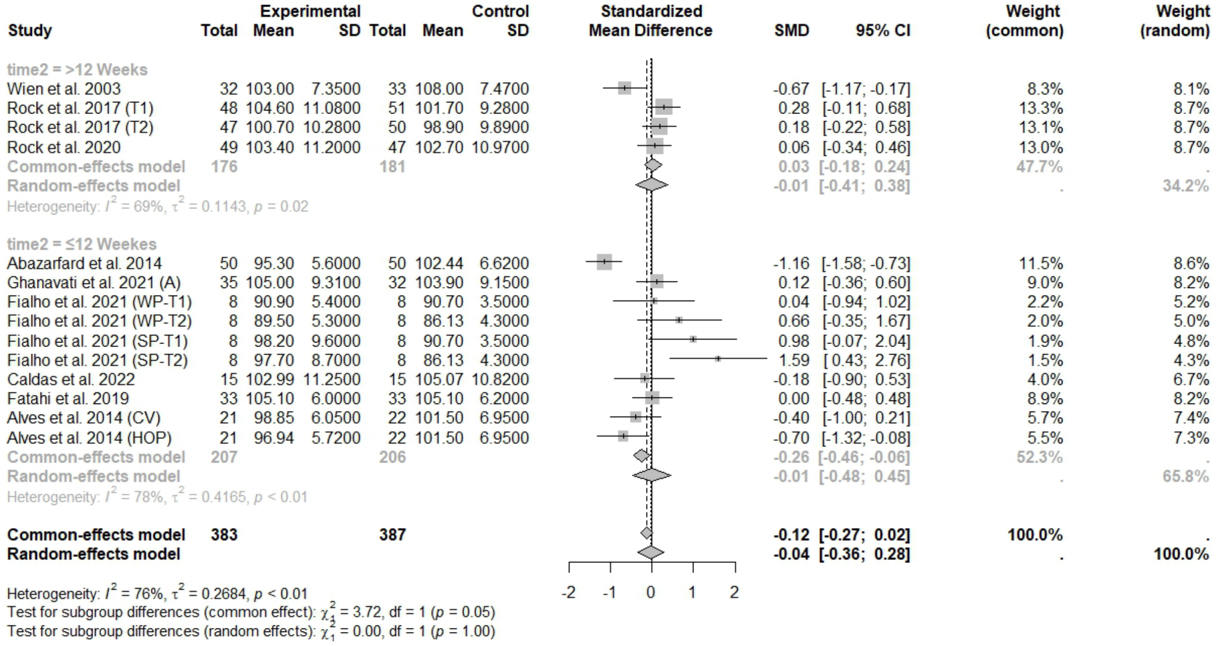

Supplementary Figure S7. Forest plot of randomized controlled trials investigating the effects of nuts consumption combined with energy-restricted diet on waist circumference according to time of intervention (≤12 and >12 weeks). Squares represent the weight of studies in meta-analysis and horizontal lines their 95 % CI; diamond's center represents the combined treatment effect and horizontal tips represent the 95 % CI. CI: confidence intervals; SMD: standard mean differences.
T1, first assessment after intervention; T2, second assessment after intervention; CV, conventional peanuts group; HOP, high-oleic peanuts group; WP, whole roasted peanuts group; SP, skinned peanuts group.

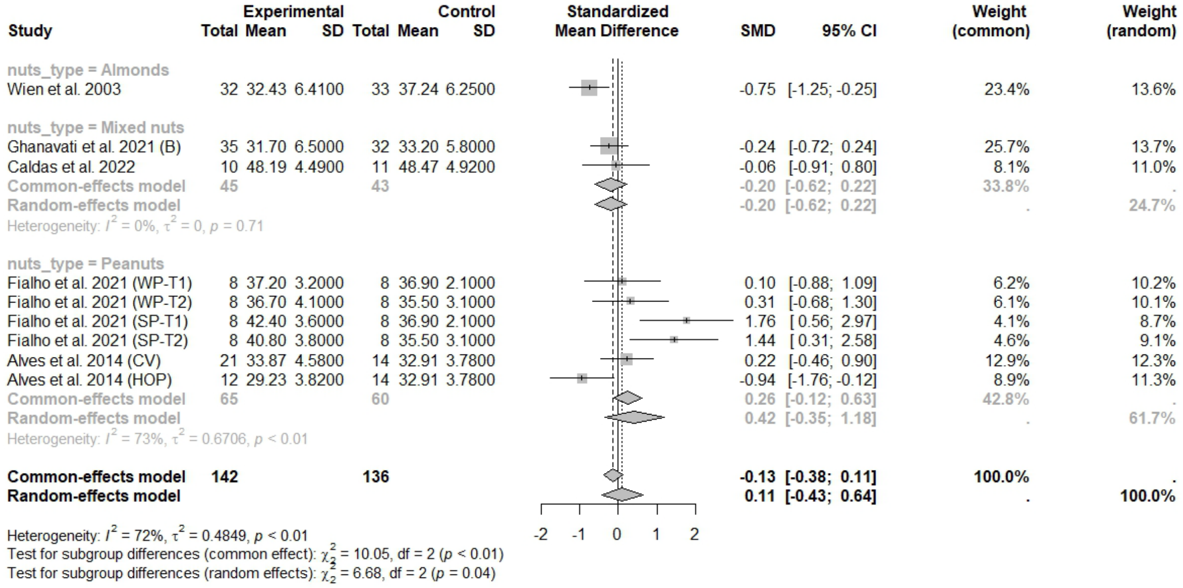

Supplementary Figure S8. Forest plot of randomized controlled trials investigating the effects of nuts consumption combined with energy-restricted diet on fat mass according to nuts type. Squares represent the weight of studies in meta-analysis and horizontal lines their 95 % CI; diamond's center represents the combined treatment effect and horizontal tips represent the 95 % CI. CI: confidence intervals; SMD: standard mean differences.

T1, first assessment after intervention; T2, second assessment after intervention; CV, conventional peanuts group; HOP, high-oleic peanuts group; WP, whole roasted peanuts group; SP, skinned peanuts group.

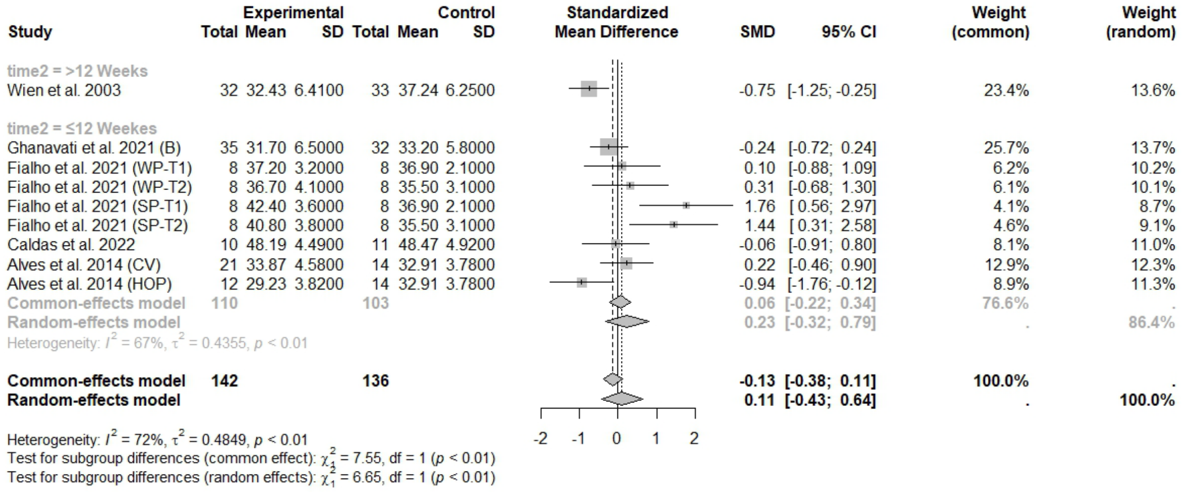

Supplementary Figure S9. Forest plot of randomized controlled trials investigating the effects of nuts consumption combined with energy-restricted diet on fat mass according to time of intervention (≤12 and >12 weeks). Squares represent the weight of studies in meta-analysis and horizontal lines their 95 % CI; diamond's center represents the combined treatment effect and horizontal tips represent the 95 % CI. CI: confidence intervals; SMD: standard mean differences.

T1, first assessment after intervention; T2, second assessment after intervention; CV, conventional peanuts group; HOP, high-oleic peanuts group; WP, whole roasted peanuts group; SP, skinned peanuts group.

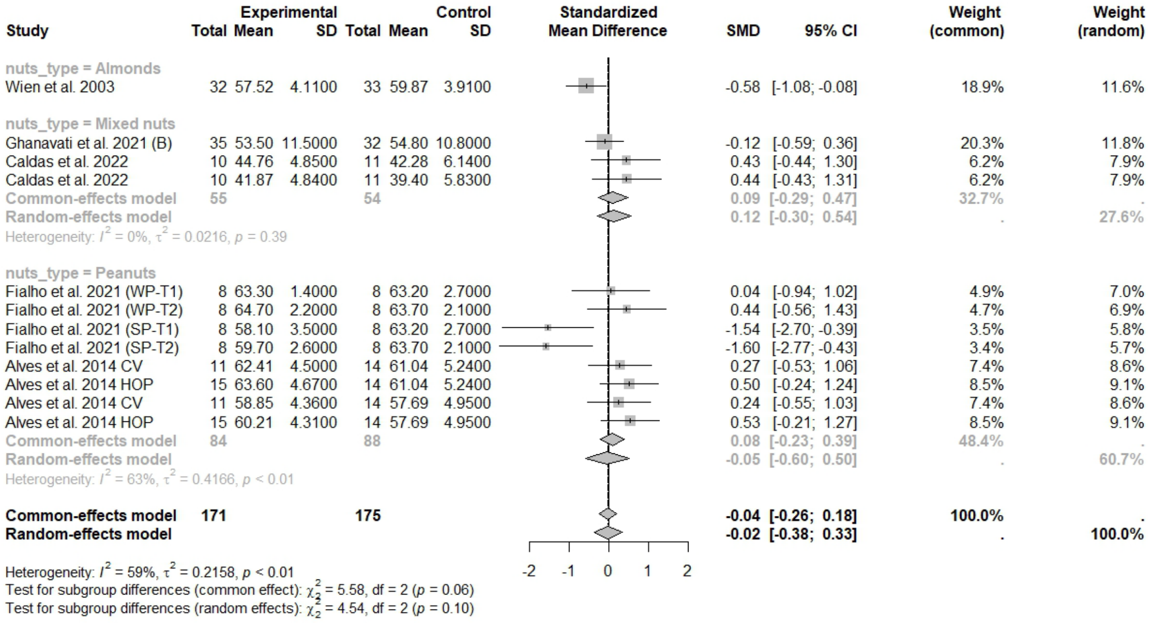

Supplementary Figure S10. Forest plot of randomized controlled trials investigating the effects of nuts consumption combined with energy-restricted diet on fat-free mass or lean mass according to nuts type. Squares represent the weight of studies in meta-analysis and horizontal lines their 95 % CI; diamond's center represents the combined treatment effect and horizontal tips represent the 95 % CI. CI: confidence intervals; SMD: standard mean differences.

T1, first assessment after intervention; T2, second assessment after intervention; CV, conventional peanuts group; HOP, high-oleic peanuts group; WP, whole roasted peanuts group; SP, skinned peanuts group.

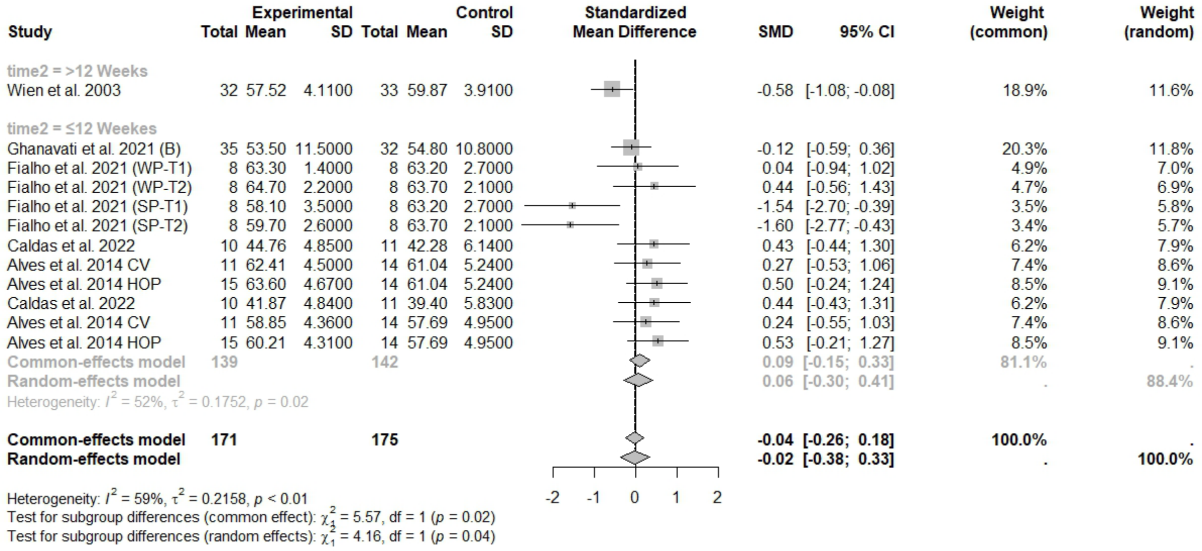

Supplementary Figure S11. Forest plot of randomized controlled trials investigating the effects of nuts consumption combined with energy-restricted diet on fat-free mass or lean mass according to time of intervention (≤12 and >12 weeks). Squares represent the weight of studies in meta-analysis and horizontal lines their 95 % CI; diamond's center represents the combined treatment effect and horizontal tips represent the 95 % CI. CI: confidence intervals; SMD: standard mean differences.

T1, first assessment after intervention; T2, second assessment after intervention; CV, conventional peanuts group; HOP, high-oleic peanuts group; WP, whole roasted peanuts group; SP, skinned peanuts group.

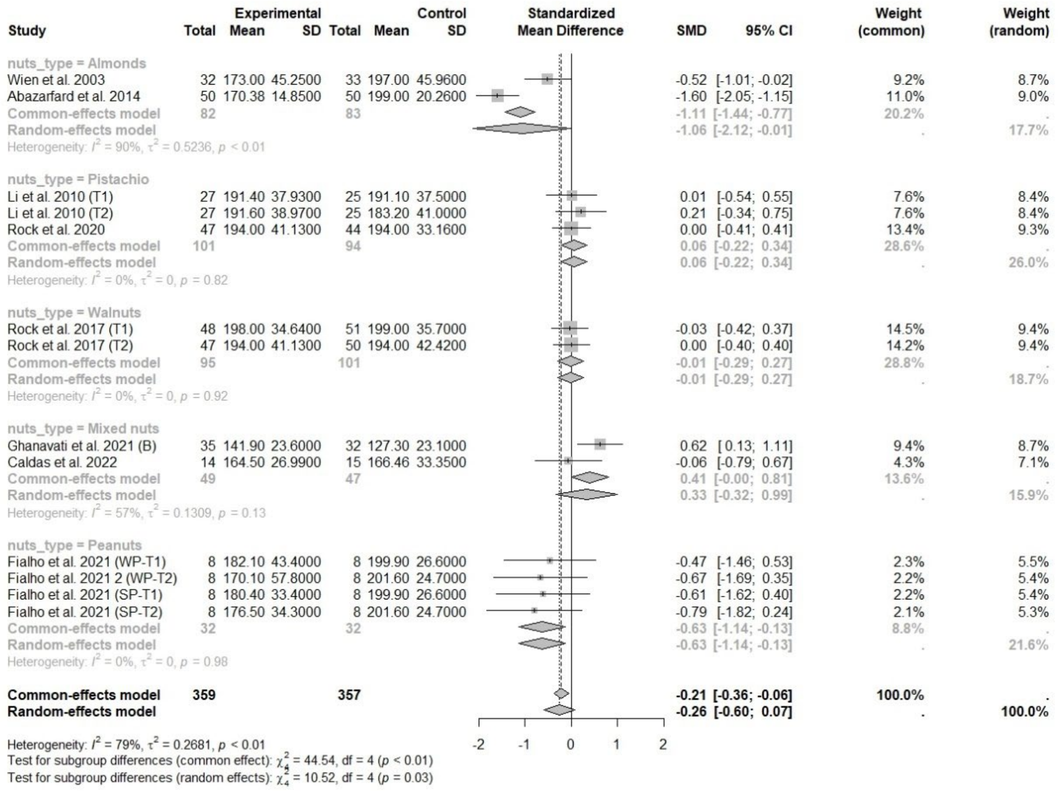

Supplementary Figure S12. Forest plot of randomized controlled trials investigating the effects of nuts consumption combined with energy-restricted diet on total cholesterol according to nuts type. Squares represent the weight of studies in meta-analysis and horizontal lines their 95 % CI; diamond's center represents the combined treatment effect and horizontal tips represent the 95 % CI. CI: confidence intervals; SMD: standard mean differences.

T1, first assessment after intervention; T2, second assessment after intervention; WP, whole roasted peanuts group; SP, skinned peanuts group.

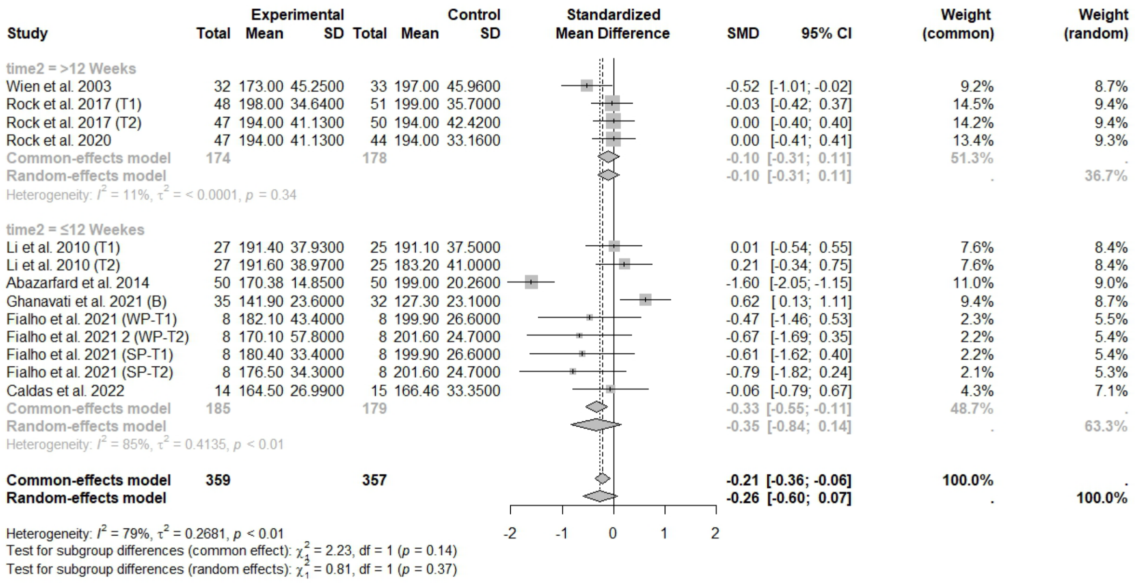

Supplementary Figure S13. Forest plot of randomized controlled trials investigating the effects of nuts consumption combined with energy-restricted diet on total cholesterol according to time of intervention (≤12 and >12 weeks). Squares represent the weight of studies in meta-analysis and horizontal lines their 95 % CI; diamond's center represents the combined treatment effect and horizontal tips represent the 95 % CI. CI: confidence intervals; SMD: standard mean differences.

T1, first assessment after intervention; T2, second assessment after intervention; WP, whole roasted peanuts group; SP, skinned peanuts group.

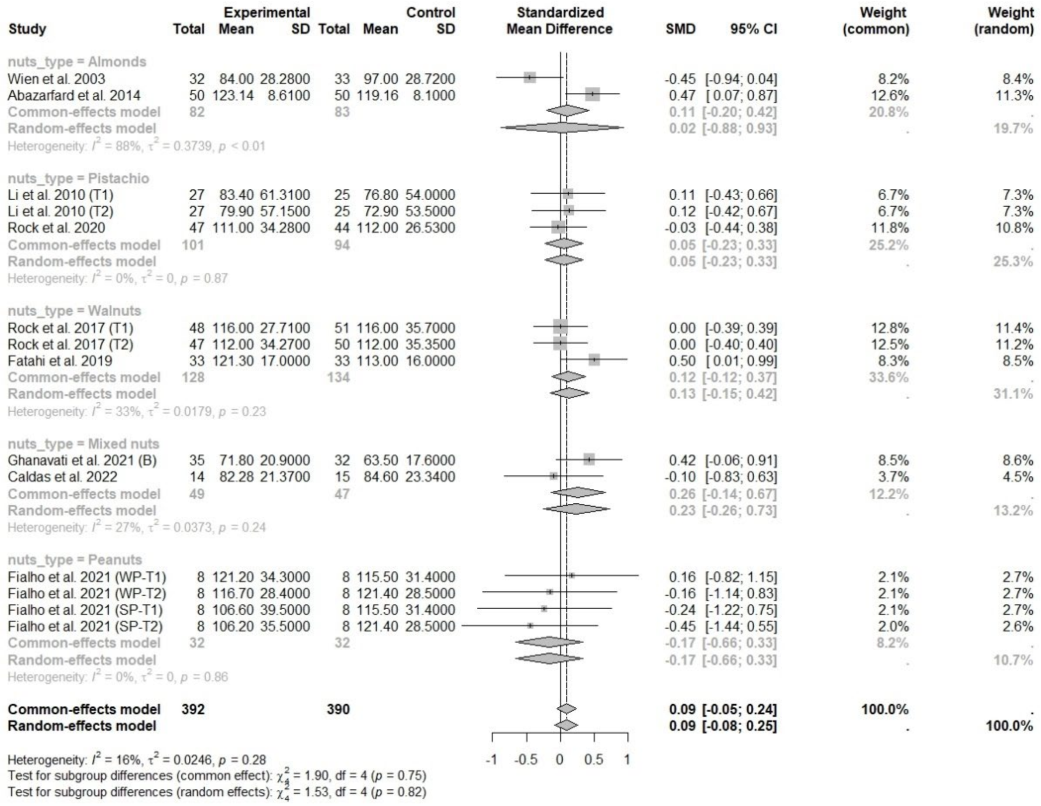

Supplementary Figure S14. Forest plot of randomized controlled trials investigating the effects of nuts consumption allied to energy-restricted diet on low-density lipoprotein cholesterol according to nuts type. Squares represent the weight of studies in meta-analysis and horizontal lines their 95 % CI; diamond's center represents the combined treatment effect and horizontal tips represent the 95 % CI. CI: confidence intervals; SMD: standard mean differences.

T1, first assessment after intervention; T2, second assessment after intervention; WP, whole roasted peanuts group; SP, skinned peanuts group.

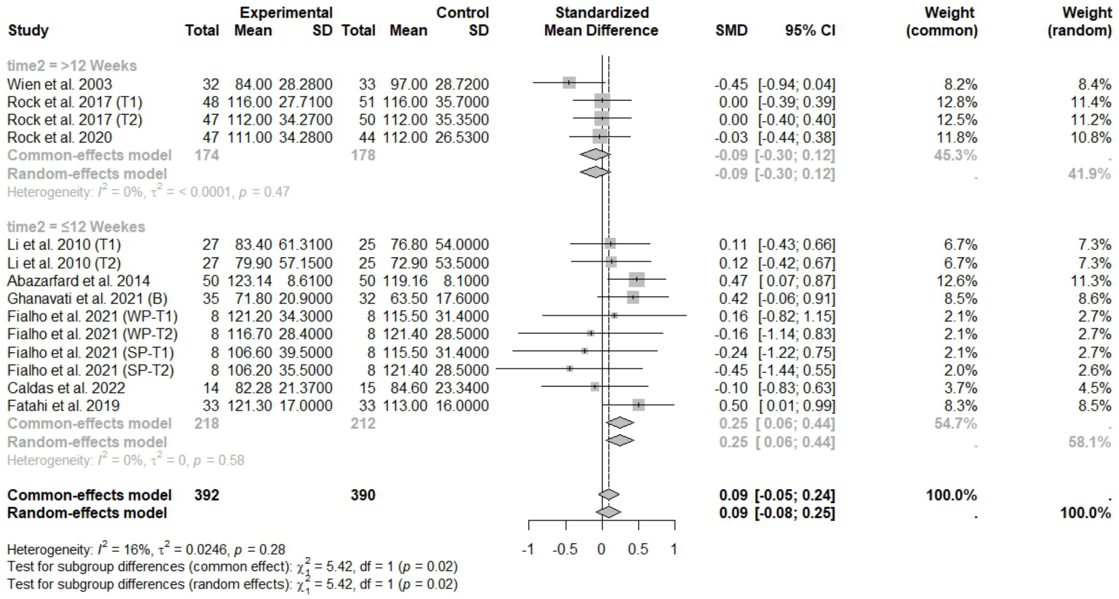

Supplementary Figure S15. Forest plot of randomized controlled trials investigating the effects of nuts consumption combined with energy-restricted diet on low-density lipoprotein cholesterol according to intervention time (≤12 and >12 weeks). Squares represent the weight of studies in meta-analysis and horizontal lines their 95 % CI; diamond's center represents the combined treatment effect and horizontal tips represent the 95 % CI. CI: confidence intervals; SMD: standard mean differences.

T1, first assessment after intervention; T2, second assessment after intervention; WP, whole roasted peanuts group; SP, skinned peanuts group.

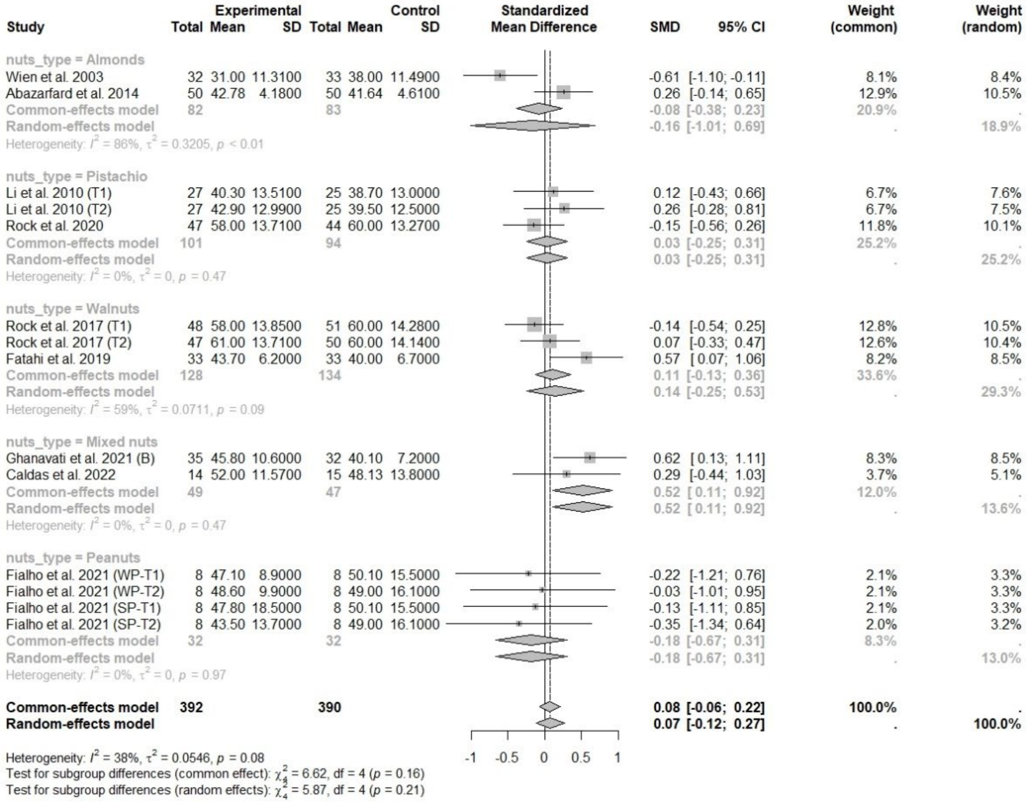

Supplementary Figure S16. Forest plot of randomized controlled trials investigating the effects of nuts consumption al combined with lied to energy-restricted diet on high-density lipoprotein cholesterol according to nuts types. Squares represent the weight of studies in meta-analysis and horizontal lines their 95 % CI; diamond's center represents the combined treatment effect and horizontal tips represent the 95 % CI. CI: confidence intervals; SMD: standard mean differences.

T1, first assessment after intervention; T2, second assessment after intervention; WP, whole roasted peanuts group; SP, skinned peanuts group.

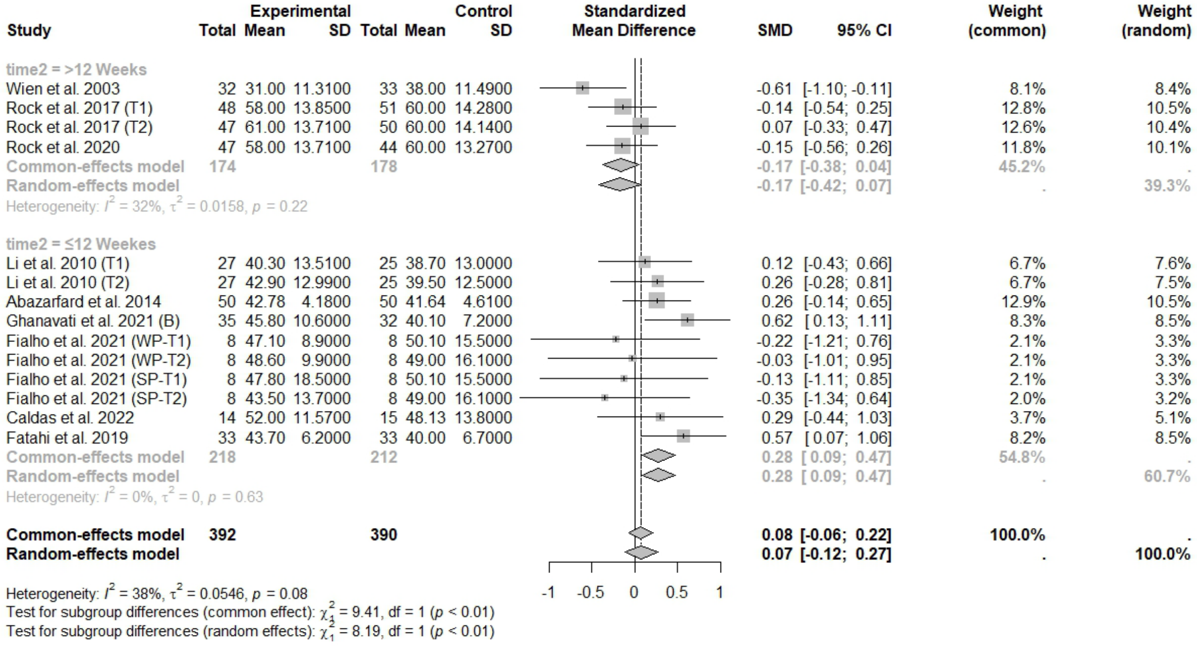

Supplementary Figure S17. Forest plot of randomized controlled trials investigating the effects of nuts consumption combined with energy-restricted diet on high-density lipoprotein cholesterol according to intervention time (≤12 and >12 weeks). Squares represent the weight of studies in meta-analysis and horizontal lines their 95 % CI; diamond's center represents the combined treatment effect and horizontal tips represent the 95 % CI. CI: confidence intervals; SMD: standard mean differences.
T1, first assessment after intervention; T2, second assessment after intervention; WP, whole roasted peanuts group; SP, skinned peanuts group.

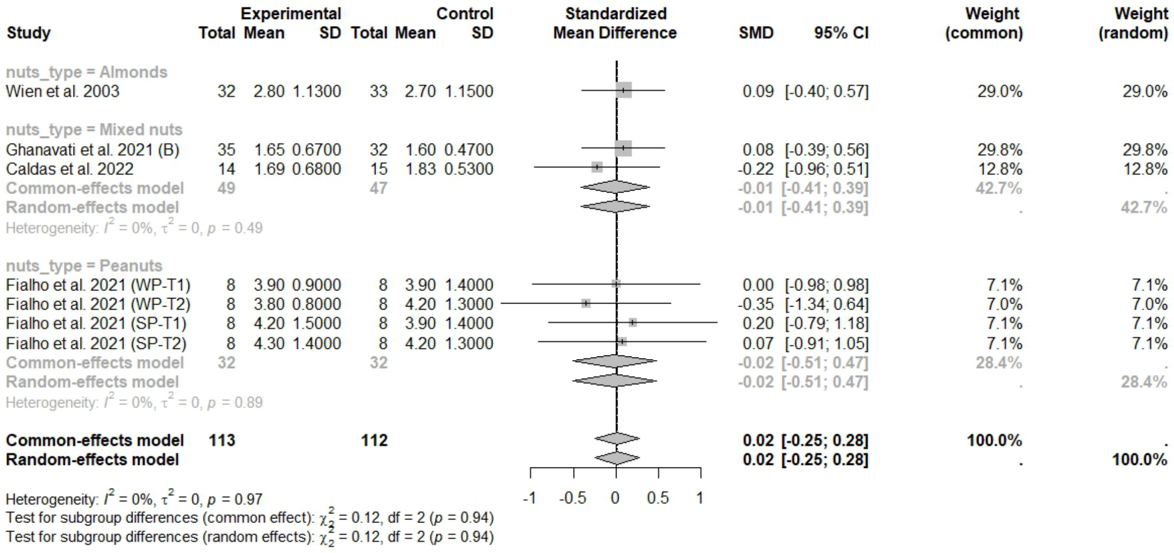

Supplementary Figure S18. Forest plot of randomized controlled trials investigating the effects of nuts consumption combined with energy-restricted diet on low-density lipoprotein cholesterol/high-density lipoprotein cholesterol ratio (LDL/HDL ratio) according to nuts type. Squares represent the weight of studies in meta-analysis and horizontal lines their 95 % CI; diamond's center represents the combined treatment effect and horizontal tips represent the 95 % CI. CI: confidence intervals; SMD: standard mean differences.

T1, first assessment after intervention; T2, second assessment after intervention; WP, whole roasted peanuts group; SP, skinned peanuts group.

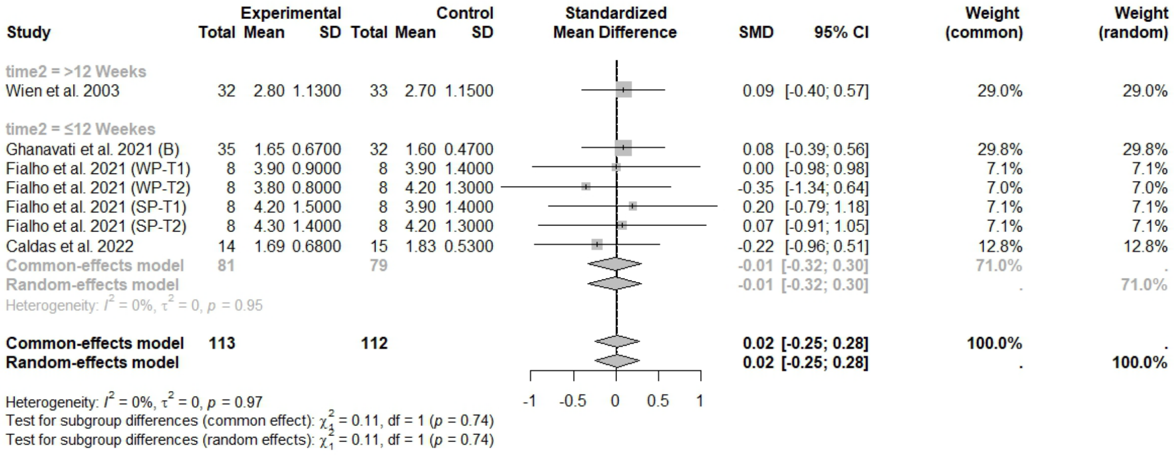

Supplementary Figure S19. Forest plot of randomized controlled trials investigating the effects of nuts consumption combined with energy-restricted diet on low-density lipoprotein cholesterol/high-density lipoprotein cholesterol ratio (LDL/HDL ratio) according to intervention time (≤12 and >12 weeks). Squares represent the weight of studies in meta-analysis and horizontal lines their 95 % CI; diamond’s center represents the combined treatment effect and horizontal tips represent the 95 % CI. CI: confidence intervals; SMD: standard mean differences.
T1, first assessment after intervention; T2, second assessment after intervention; WP, whole roasted peanuts group; SP, skinned peanuts group.

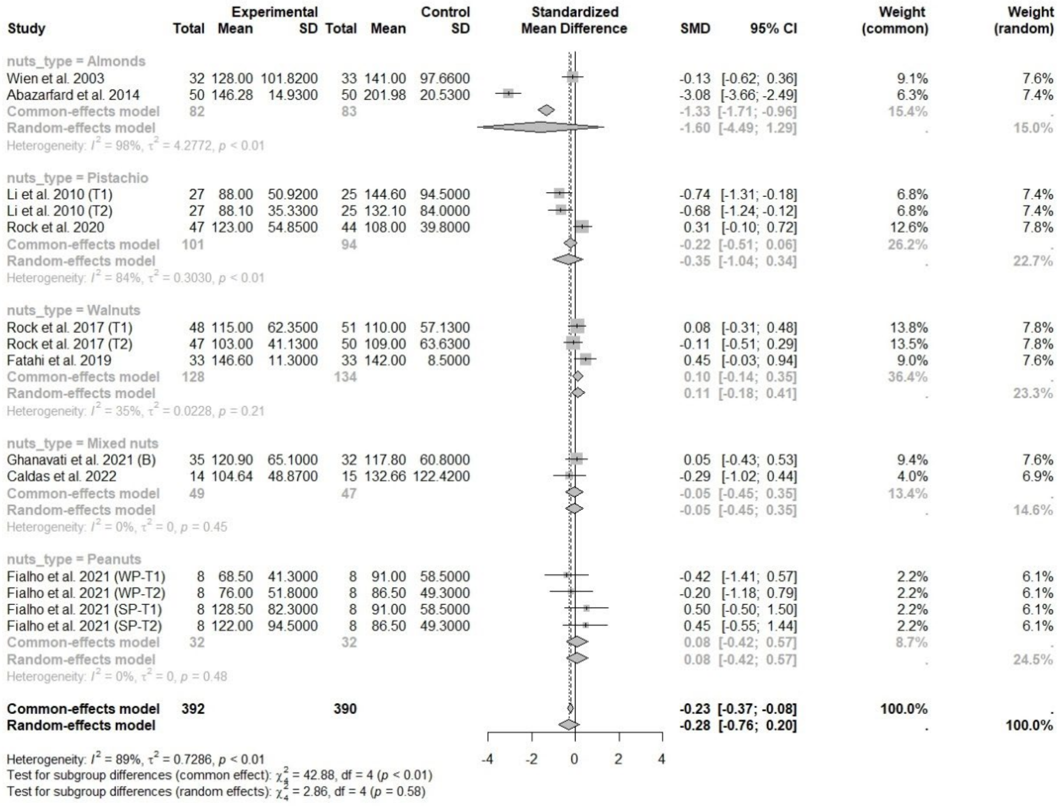

Supplementary Figure S20. Forest plot of randomized controlled trials investigating the effects of nuts consumption combined with energy-restricted diet on triglyceride levels according to nuts type. Squares represent the weight of studies in meta-analysis and horizontal lines their 95 % CI; diamond’s center represents the combined treatment effect and horizontal tips represent the 95 % CI. CI: confidence intervals; SMD: standard mean differences.
T1, first assessment after intervention; T2, second assessment after intervention; WP, whole roasted peanuts group; SP, skinned peanuts group.

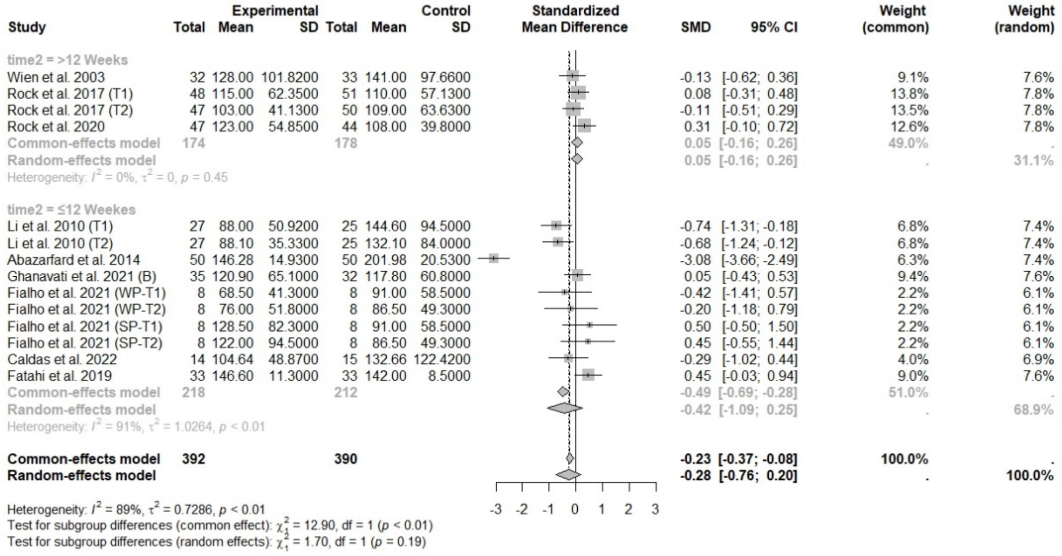

Supplementary Figure S21. Forest plot of randomized controlled trials investigating the effects of nuts consumption combined with energy-restricted diet on triglyceride levels according to intervention time (≤12 and >12 weeks). Squares represent the weight of studies in meta-analysis and horizontal lines their 95 % CI; diamond's center represents the combined treatment effect and horizontal tips represent the 95 % CI. CI: confidence intervals; SMD: standard mean differences.

T1, first assessment after intervention; T2, second assessment after intervention; WP, whole roasted peanuts group; SP, skinned peanuts group.

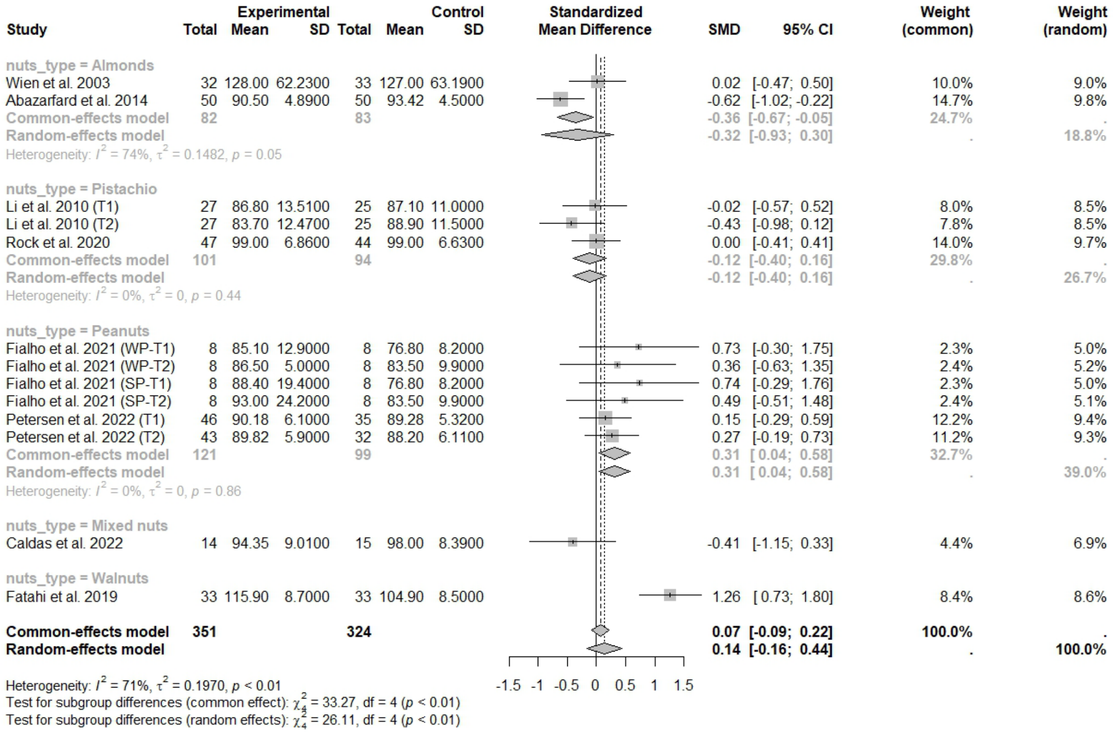

Supplementary Figure S22. Forest plot of randomized controlled trials investigating the effects of nuts consumption combined with energy-restricted diet on fasting glucose levels according to nuts type. Squares represent the weight of studies in meta-analysis and horizontal lines their 95 % CI; diamond's center represents the combined treatment effect and horizontal tips represent the 95 % CI. CI: confidence intervals; SMD: standard mean differences.

T1, first assessment after intervention; T2, second assessment after intervention; WP, whole roasted peanuts group; SP, skinned peanuts group.

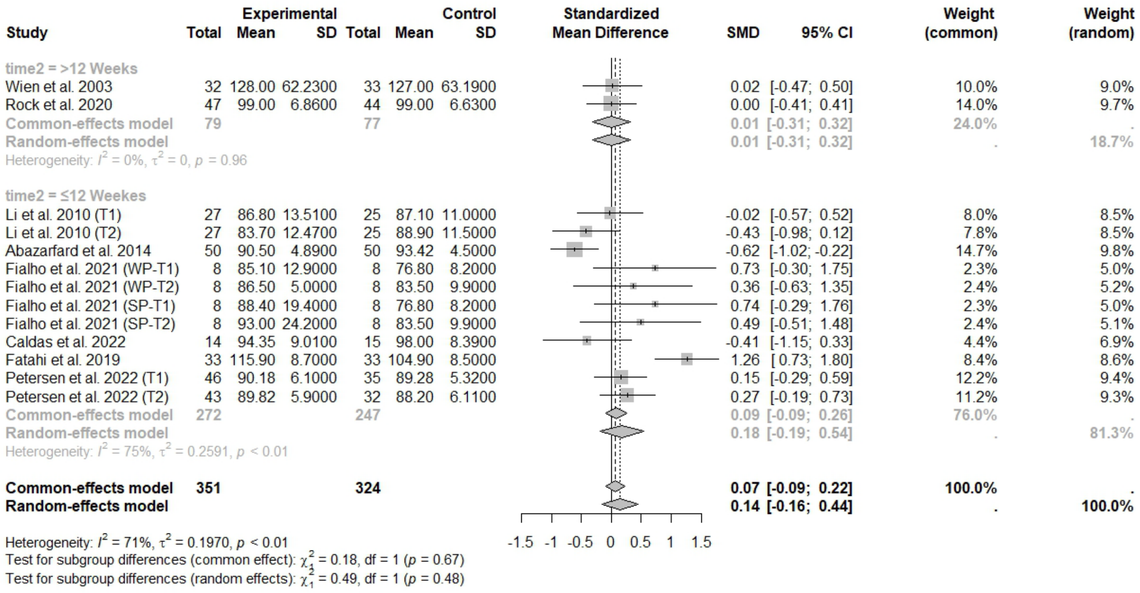

Supplementary Figure S23. Forest plot of randomized controlled trials investigating the effects of nuts consumption combined with energy-restricted diet on fasting glucose levels according to intervention time (≤12 and >12 weeks). Squares represent the weight of studies in meta-analysis and horizontal lines their 95 % CI; diamond's center represents the combined treatment effect and horizontal tips represent the 95 % CI. CI: confidence intervals; SMD: standard mean differences.
T1, first assessment after intervention; T2, second assessment after intervention; WP, whole roasted peanuts group; SP, skinned peanuts group.

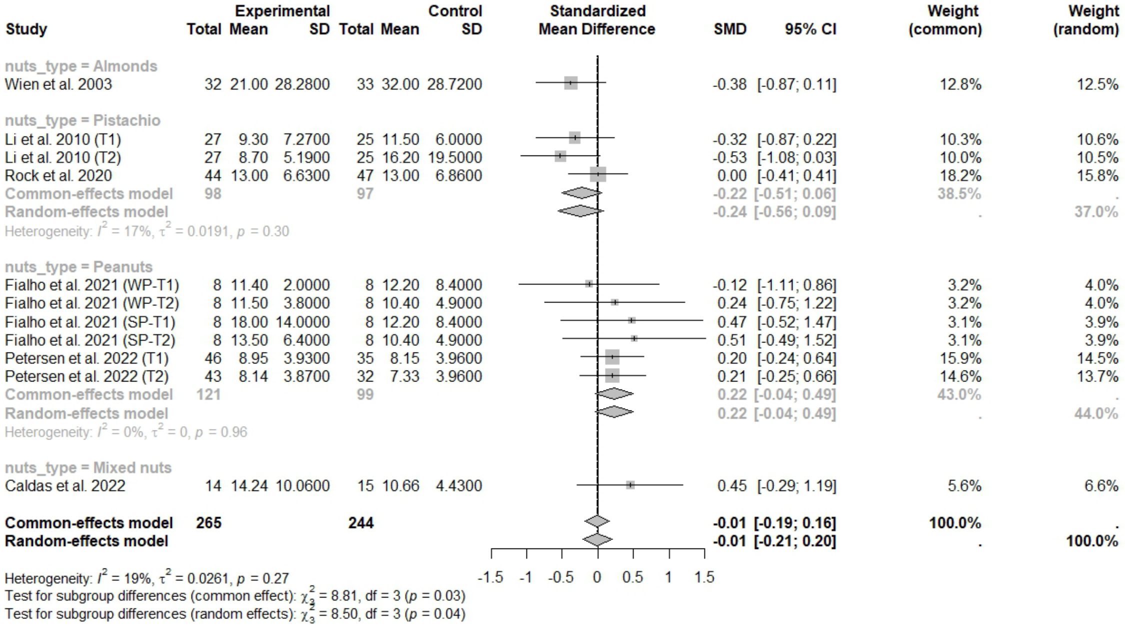

Supplementary Figure S24. Forest plot of randomized controlled trials investigating the effects of nuts consumption combined with energy-restricted diet on fasting insulin levels according to nuts type. Squares represent the weight of studies in meta-analysis and horizontal lines their 95 % CI; diamond's center represents the combined treatment effect and horizontal tips represent the 95 % CI. CI: confidence intervals; SMD: standard mean differences.

T1, first assessment after intervention; T2, second assessment after intervention; WP, whole roasted peanuts group; SP, skinned peanuts group.

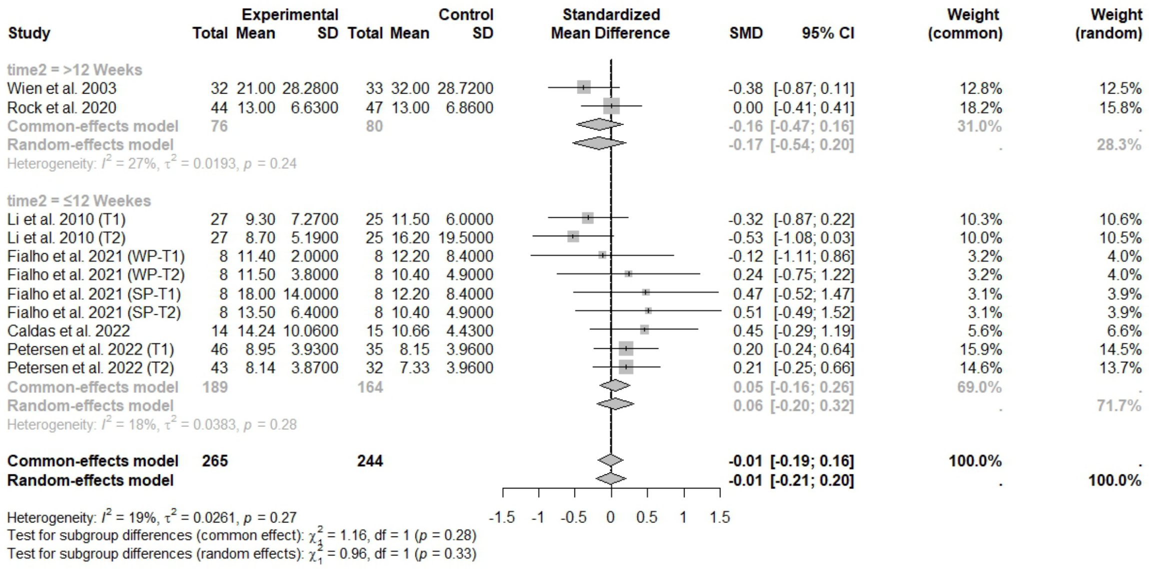

Supplementary Figure S25. Forest plot of randomized controlled trials investigating the effects of nuts consumption combined with energy-restricted diet on fasting insulin levels according to intervention time (≤12 and >12 weeks). Squares represent the weight of studies in meta-analysis and horizontal lines their 95 % CI; diamond's center represents the combined treatment effect and horizontal tips represent the 95 % CI. CI: confidence intervals; SMD: standard mean differences.

T1, first assessment after intervention; T2, second assessment after intervention; WP, whole roasted peanuts group; SP, skinned peanuts group.

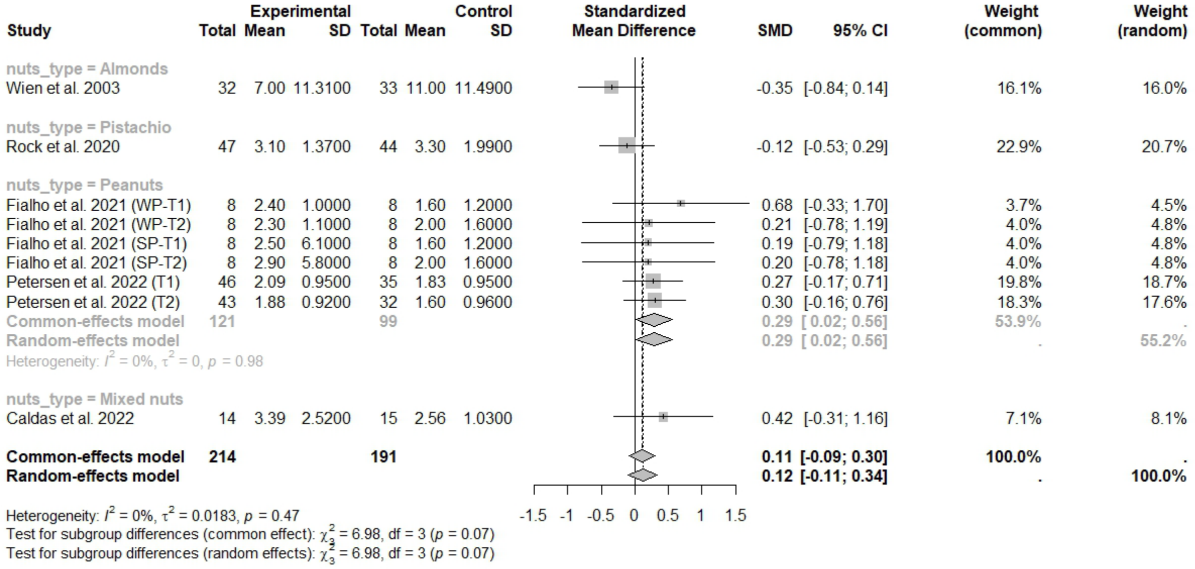

Supplementary Figure S26. Forest plot of randomized controlled trials investigating the effects of nuts consumption combined with energy-restricted diet on HOMA-IR according to nuts type. Squares represent the weight of studies in meta-analysis and horizontal lines their 95 % CI; diamond's center represents the combined treatment effect and horizontal tips represent the 95 % CI. CI: confidence intervals; SMD: standard mean differences. T1, first assessment after intervention; T2, second assessment after intervention; WP, whole roasted peanuts group; SP, skinned peanuts group.

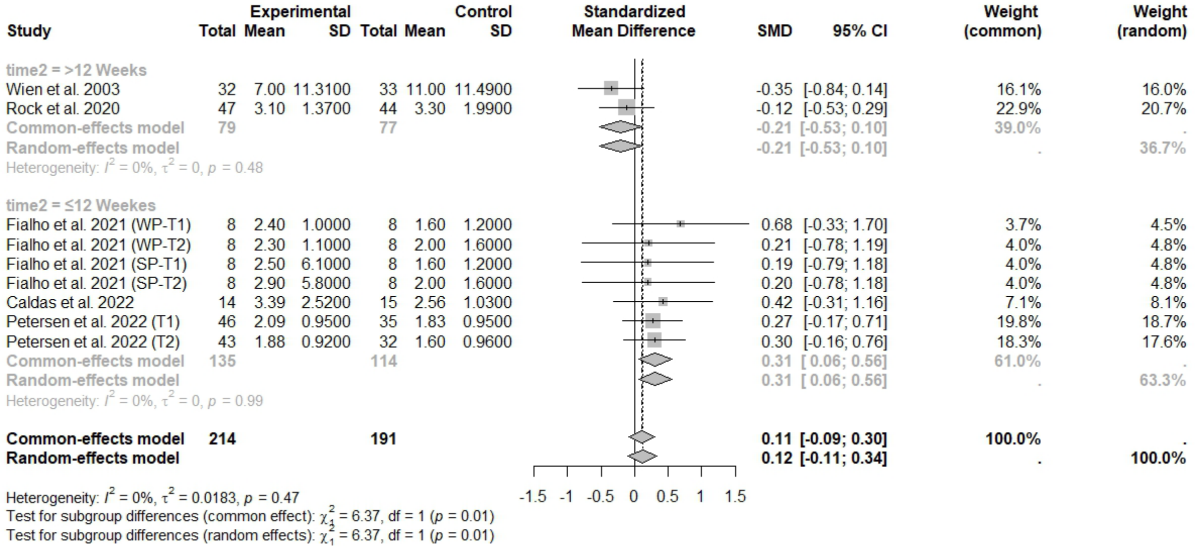

Supplementary Figure S27. Forest plot of randomized controlled trials investigating the effects of nuts consumption combined with energy-restricted diet on HOMA-IR

according to intervention time (≤12 and >12 weeks). Squares represent the weight of studies in meta-analysis and horizontal lines their 95 % CI; diamond's center represents the combined treatment effect and horizontal tips represent the 95 % CI. CI: confidence intervals; SMD: standard mean differences.
T1, first assessment after intervention; T2, second assessment after intervention; WP, whole roasted peanuts group; SP, skinned peanuts group.

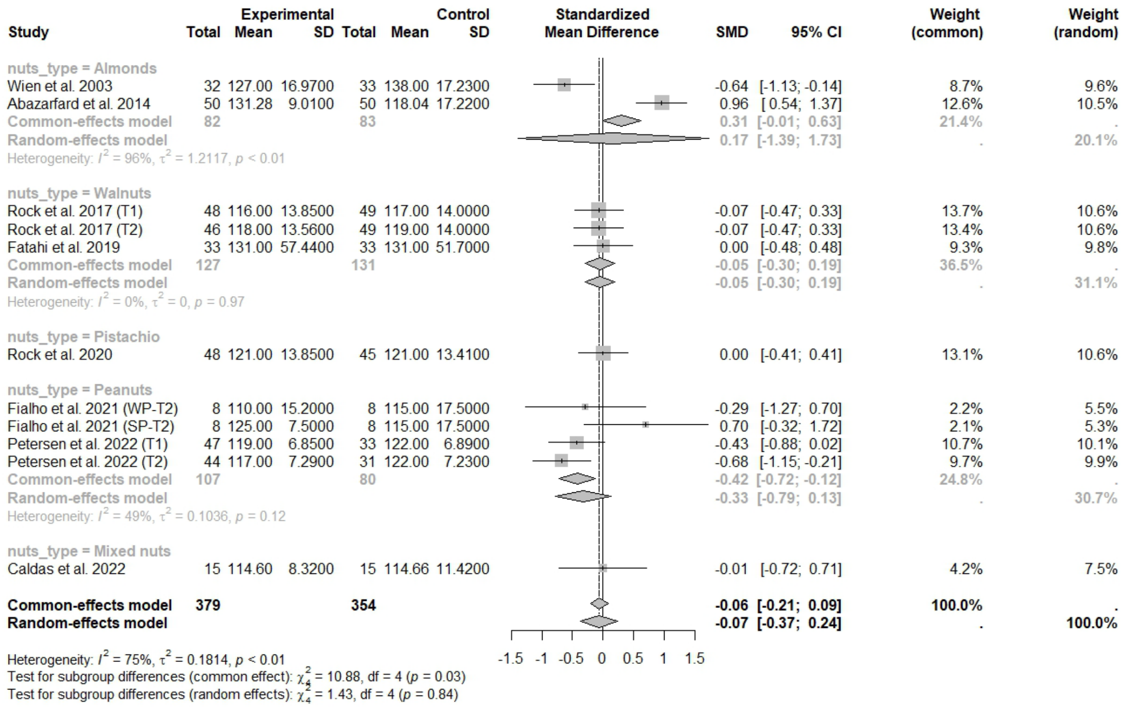

Supplementary Figure S28. Forest plot of randomized controlled trials investigating the effects of nuts consumption combined with energy-restricted diet on systolic blood pressure according to nuts type. Squares represent the weight of studies in meta-analysis and horizontal lines their 95 % CI; diamond's center represents the combined treatment effect and horizontal tips represent the 95 % CI. CI: confidence intervals; SMD: standard mean differences. T1, first assessment after intervention; T2, second assessment after intervention; WP, whole roasted peanuts group; SP, skinned peanuts group.

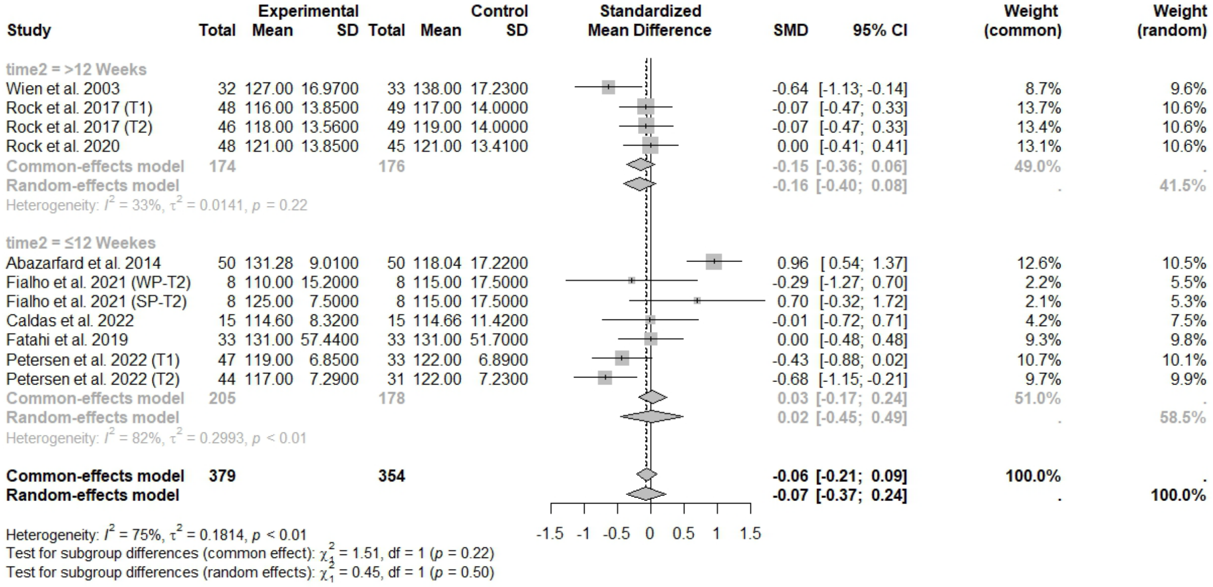

Supplementary Figure S29. Forest plot of randomized controlled trials investigating the effects of nuts consumption combined with energy-restricted diet on systolic blood pressure according to intervention time (≤12 and >12 weeks). Squares represent the weight of studies in meta-analysis and horizontal lines their 95 % CI; diamond's center represents the combined treatment effect and horizontal tips represent the 95 % CI. CI: confidence intervals; SMD: standard mean differences. T1, first assessment after intervention; T2, second assessment after intervention; WP, whole roasted peanuts group; SP, skinned peanuts groups

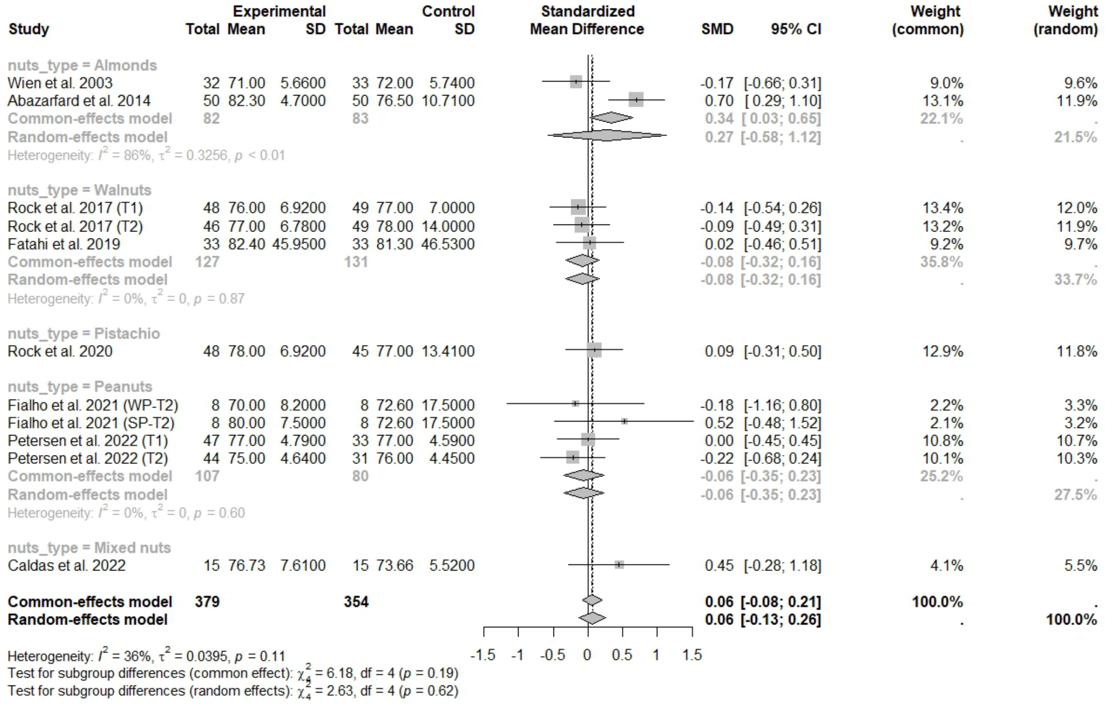

Supplementary Figure S30. Forest plot of randomized controlled trials investigating the effects of nuts consumption combined with energy-restricted diet on diastolic blood pressure according to nuts type. Squares represent the weight of studies in meta-analysis and horizontal lines their 95 % CI; diamond's center represents the combined treatment effect and horizontal tips represent the 95 % CI. CI: confidence intervals; SMD: standard mean differences.

T1, first assessment after intervention; T2, second assessment after intervention; WP, whole roasted peanuts group; SP, skinned peanuts group.

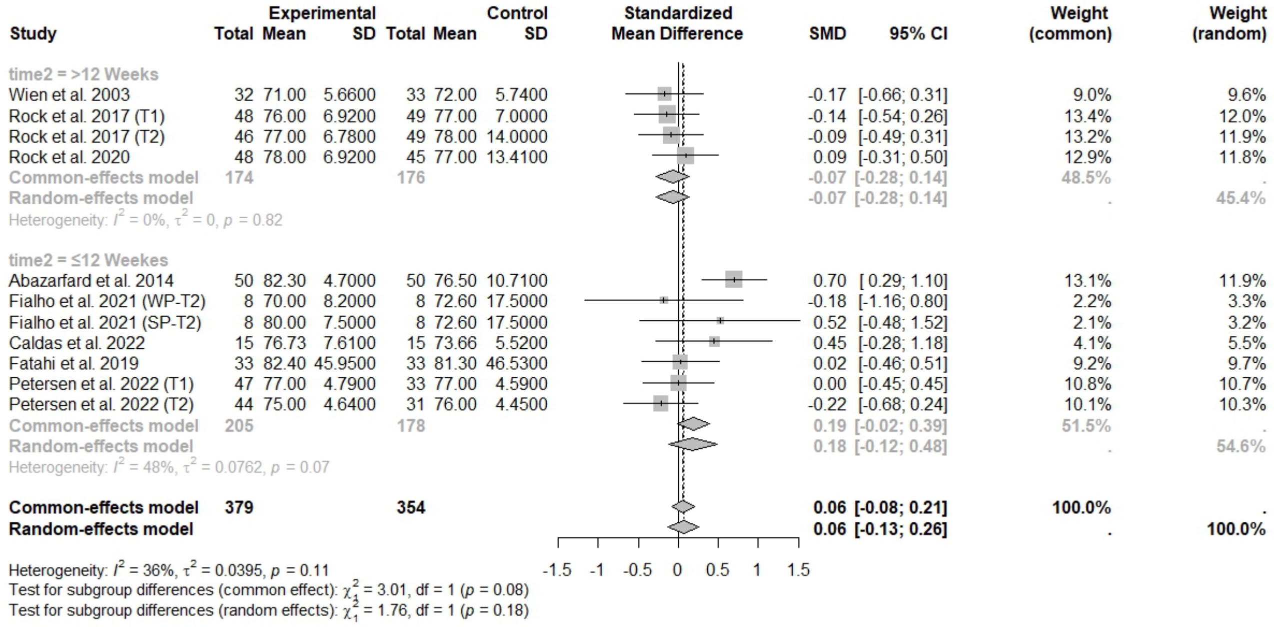

Supplementary Figure S31. Forest plot of randomized controlled trials investigating the effects of nuts consumption combined with energy-restricted diet on diastolic blood pressure according to intervention time (≤12 and >12 weeks). Squares represent the weight of studies in meta-analysis and horizontal lines their 95 % CI; diamond's center represents the combined treatment effect and horizontal tips represent the 95 % CI. CI: confidence intervals; SMD: standard mean differences. T1, first assessment after intervention; T2, second assessment after intervention; WP, whole roasted peanuts group; SP, skinned peanuts group.
